# Anticancer therapeutic potential of multimodal targeting agent- “phosphorylated galactosylated chitosan coated magnetic nanoparticles” against *N*-*nitrosodiethylamine*-induced hepatocellular carcinoma

**DOI:** 10.1007/s13346-024-01655-1

**Published:** 2024-07-11

**Authors:** Anushree Udupi, Sachin Shetty, Jesil Mathew Aranjani, Rajesh Kumar, Sanjay Bharati

**Affiliations:** 1 Department of Nuclear Medicine, Manipal College of Health Professions, Manipal Academy of Higher Education, Manipal, 576104 Karnataka India; 2 Department of Pharmaceutical Biotechnology, Manipal College of Pharmaceutical Sciences, Manipal Academy of Higher Education, Manipal, 576104 Karnataka India; 3 Department of Nuclear Medicine, All India Institute of Medical Sciences, Jodhpur, 342005 Rajasthan India

**Keywords:** Targeted drug delivery, Asialoglycoprotein receptor, Superparamagnetic iron oxide nanoparticles, NDEA, Liver cancer

## Abstract

Superparamagnetic iron oxide nanoparticles (SPIONs) are extensively used as carriers in targeted drug delivery and has several advantages in the field of magnetic hyperthermia, chemodynamic therapy and magnet assisted radionuclide therapy. The characteristics of SPIONs can be tailored to deliver drugs into tumor *via* “passive targeting” and they can also be coated with tissue-specific agents to enhance tumor uptake *via* “active targeting”. In our earlier studies, we developed HCC specific targeting agent- “phosphorylated galactosylated chitosan”(PGC) for targeting asialoglycoprotein receptors. Considering their encouraging results, in this study we developed a multifunctional targeting system- “phosphorylated galactosylated chitosan-coated magnetic nanoparticles”(PGCMNPs) for targeting HCC. PGCMNPs were synthesized by co-precipitation method and characterized by DLS, XRD, TEM, VSM, elemental analysis and FT-IR spectroscopy. PGCMNPs were evaluated for in vitro antioxidant properties, uptake in HepG2 cells, biodistribution, in vivo toxicity and were also evaluated for anticancer therapeutic potential against NDEA-induced HCC in mice model in terms of tumor status, electrical properties, antioxidant defense status and apoptosis. The characterization studies confirmed successful formation of PGCMNPs with superparamagnetic properties. The internalization studies demonstrated (99–100)% uptake of PGCMNPs in HepG2 cells. These results were also supported by biodistribution studies in which increased iron content (296%) was noted inside the hepatocytes. Further, PGCMNPs exhibited no in vivo toxicity. The anticancer therapeutic potential was evident from observation that PGCMNPs treatment decreased tumor bearing animals (41.6%) and significantly (p ≤ 0.05) lowered tumor multiplicity. Overall, this study indicated that PGCMNPs with improved properties are efficiently taken-up by hepatoma cells and has therapeutic potential against HCC. Further, this agent can be tagged with ^32^P and hence can offer multimodal cancer treatment options *via* radiation ablation as well as magnetic hyperthermia.

**Graphical Abstract:**

Schematic illustration of PGCMNPs synthesis, characterization and its anticancer potential: PGCMNPs were synthesized by co-precipitation method. The successful synthesis of PGCMNPs was confirmed by physical and chemical characterizations. PGCMNPs were biocompatible and exhibited no toxicity at tested parameters. PGCMNPs demonstrated higher uptake in HepG2 cells. The anticancer therapeutic potential of PGCMNPs in HCC mouse model, was evident from improved tumor statistics, increased low conductivity tumors and increased apoptosis mediated cell death.

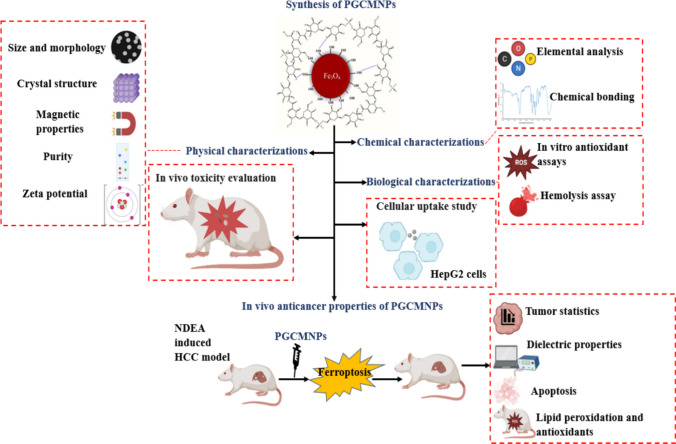

**Supplementary Information:**

The online version contains supplementary material available at 10.1007/s13346-024-01655-1.

## Introduction

Targeted cancer therapies or precision medicine is a treatment strategy which precisely delivers chemotherapeutic drugs into specific tissue or organ of interest [1]. Unlike conventional chemotherapy, targeted cancer therapies are capable of distinguishing cancer cells from the normal, based on cellular/tissue composition, architecture or microenvironment [2]. Targeting of the drugs can be achieved using different carrier systems [3]. The physicochemical characteristics of the carrier systems can be modified to deliver the drugs into tumor via “passive or active mechanisms”. In the case of passive targeting, the drug molecules preferably gets accumulated in the favorable tumor microenvironment due to “enhanced permeability and retention effect” (EPR) [4]. In active targeting, the drug molecules are delivered based on specific interaction of the biomolecules present on the drug-carrier and the target tumor tissue [5]. The active targeting can be achieved further through mechanisms like inverse targeting (suppression of reticuloendothelial uptake and defense mechanisms), ligand-mediated targeting (targeting the receptors on the tumor via ligands on the carrier system), physical targeting (targeting the drug molecules via external physical stimuli like temperature, electrical field or magnets) and dual targeting (drug delivery system in which both carrier and the drug acts as therapeutic agents) [6]. Additional advantage of targeting system is its minimal invasive nature as it does not require direct tumoral injection [7]. Therefore, different carrier systems are now the current area of interest in various cancers like colorectal cancer [8], breast cancer [9], liver cancer [10], lung cancer [11], esophageal cancer [12] and many others [13, 14].

Superparamagnetic iron oxide nanoparticles (SPIONs) are extensively used as carriers in targeted drug delivery, magnetic hyperthermia, chemodynamic therapy, and magnetic field assisted radionuclide therapy etc. [15, 16]. SPIONs show intrinsic property of accumulation within the tumor parenchyma which is mediated through “EPR” effect. The tumor accumulation can be further enhanced by coating SPIONs with tissue specific targeting agents without altering the properties of SPIONs [17]. In our previous study, we developed and studied the biological properties of HCC specific targeting agent “phosphorylated galactosylated chitosan (PGC)”. PGC was designed to target hepatoma cells *via* active targeting mediated through asialoglycoprotein receptor (ASGPRs) [18]. ASGPR is the most explored or common target receptor in hepatocellular carcinoma (HCC) due to its high expression (76,000 ASGPRs/ cell) specifically on the membranes of hepatoma cells [19, 20]. The results showed good targeting of PGC into the hepatoma cells (active targeting) as well as anticancer therapeutic effect against HCC (dual targeting) [18]. Considering this, in the present study we aimed to develop a targeting system which can be used for both effective targeting as well as multimodal therapy of HCC.

## Materials and methods

### Chemicals and kits

*N-Nitrosodiethylamine* (NDEA) was procured from Sigma Aldrich Co. (St. Louis, USA). Ferric chloride, ferrous chloride, ammonium hydroxide were obtained from Sisco Research Laboratories Pvt. Ltd. (Mumbai, India). Kits for the estimation of liver and kidney injury markers were obtained from Coral clinical systems (Goa, India). Terminal de-oxy-nucleotidyl transferase “(TdT) dUTP Nick-End Labeling” (TUNEL assay) kit was procured from Trevigen (Gaithersburg, Maryland, United States). All the other chemicals used in the study were of prime quality and analytical grade.

### Preparation of phosphorylated galactosylated chitosan coated magnetic nanoparticles (PGCMNPs)

PGCMNPs were synthesized by the method of co-precipitation using four-necked glass balloon apparatus (supplementary data 1). Briefly, FeCl_3_ and FeCl_2_ (2:1 in double distilled water, 100 mL) were taken in a glass balloon apparatus and heated at 80 °C for 20 minutes under constant stirring (2500 rpm). Ammonium hydroxide (32%, 15 mL) was then added slowly into the glass apparatus (1 drop/30 s). After the addition of 2 mL of ammonium hydroxide, (PGC) (4 g in 10 mL of) was added dropwise and the solution was stirred for 1 hour. The preparative methods of PGC were described in our earlier study [18, 21]. The entire procedure of PGCMNPs synthesis was carried out in the presence of continuous supply of N_2_ gas. PGCMNPs were then separated from the colloidal solution using magnetic decantation.

### Characterization of PGCMNPs

#### Physical characterizations

##### Hydrodynamic size (Dynamic light scattering)

Hydrodynamic size of PGCMNPs in water/biological solutions was crucial for its cellular uptake. Therefore, hydrodynamic size of PGCMNPs were determined using dynamic light scattering analysis at 25 °C using dynamic light scattering analyzer (Malvern Instruments Ltd., Worcestershire, UK). Samples were prepared in deionized water (1 mg/mL) and measured (n=3, 12 runs each) in a disposable cuvette. Results obtained were expressed as Mean ± SD.

##### Surface charge (ζ -potential analysis)

Surface charge is an important parameter which determines the stability of a nanoparticle in biological fluids and cellular membranes. Surface charge on PGCMNPs was determined using zeta potential analyzer (Malvern Instruments Ltd., Worcestershire, United Kingdom). Samples were prepared in deionized water (2 mg/mL) and measured (n=3, 12 runs each) in a transparent cuvette at 25 °C. The scattered light detection was fixed at a 90° backscatter angle. The final results obtained were presented as Mean ± SD.

##### Crystal structure (X-ray diffraction analysis)

X-ray diffraction analysis (XRD) of PGCMNPs was carried out to determine the properties of iron ore crystals like crystal type, size, composition and phases, which are critical for its biological actions. XRD of PGCMNPs was carried out using X-ray diffraction analysis equipment (Rigaku ultima IV, Tokyo, Japan) with a copper source of Cu -α at 1.540562 Å (Filter: Ni) and scan rate of 2 degree/minute. Data obtained were normalized on a scale of (0 to 100), baselined and compared with standard magnetite (JCPDS card no. 82-1533). The spectra were analyzed for all the phases of magnetite and crystalline size was determined.

##### Size and morphology (Transmission *electron* microscopy)

Determination of physical properties like shape, core size, agglomeration and atomic arrangements in crystal were important for PGCMNPs behavior in vivo. Therefore, transmission electron microscopy of PGCMNPs was carried out using high-resolution transmission electron microscope (HR-TEM) (JEOL JEM 2100F) (Akishima, Tokyo, Japan). The HR-TEM micrographs of solid samples were obtained using field emission gun operated at an accelerating voltage of 200 kV. Data was analyzed for average core size and results were expressed as Mean ± SD. TEM micrographs were also analyzed for lattice fringes and selected area electron diffraction (SAED) pattern of crystal phases.

##### Thin layer chromatography for the determination of purity

Thin layer chromatography (TLC) was performed to determine the purity of the final compound PGCMNPs. 2 µL of sample (5 mg/mL in 50% methanol) was spotted on silica plates (Merck 60 F 254, Darmstadt, Germany). The mobile phase constituted a mixture of butanol: acetic acid and water in the ratio of 5: 1.5: 3.5 (v/v/v). Spot visualization was obtained by spraying ninhydrin solution (0.2% of w/v of ninhydrin in ethanol) and heating the plates at 110 °C for 1 minute.

##### Magnetic properties (vibrating sample magnetometry)

Vibrating sample magnetometry (VSM) was used to determine the magnetic profile of PGCMNPs essential for its extended therapeutic applications. VSM was performed using vibrating sample magnetometer (Lake Shore cryotronics, Westerville, United States). Lyophilized sample (100 mg) was placed in the sample holder tube and was magnetized in the uniform magnetic field (1.5 T) created by external electromagnetics at room temperature. The saturation curve obtained was then analyzed for magnetic saturation, retentivity and coercivity.

#### Chemical characterizations

##### Elemental analysis

Elemental constituents (percentage of C, N, O and P) in PGCMNPs were analyzed using scanning electron microscopy-energy dispersive X-ray spectroscopy (SEM-EDS) (JEOL, Tokyo, Japan). Briefly, lyophilized samples were mounted on the sample holder using double-sided conductive carbon tapes and sputtered with Ag for elemental analysis. Throughout the experiment, the air was conditioned at 21-24 °C and relative humidity was maintained as 60%. Signals produced in the SEM-EDS system were used for elemental quantification.

##### Fourier transform infrared spectroscopy

In order to determine the surface coating of PGC onto iron oxide nanoparticles, Fourier transform infrared spectroscopy (FT-IR) of PGCMNPs was obtained using an FT-IR spectrophotometer (FT-IR- 8300, Shimadzu, Japan). The lyophilized PGCMNPs were mixed with KBr (Infra-red inactive) to form pellet and read using FT-IR spectrometer in transmission mode (4000-500 cm^-1^). Datum obtained was then corrected for background transmittance, baselined, normalized and analyzed for the characteristic peaks.

#### Biological characterizations

##### In vitro antioxidant assays

In vitro antioxidant properties like ferric ion reducing ability, metal chelating potential, superoxide radical (SOR) inhibiting ability, lipid peroxide (LPO) inhibiting ability of PGCMNPs were determined at different concentrations (0.25, 0.5 and 1 mg).

Ferric ion reducing ability was based on the ability of the compound to reduce ferric ions to ferrous ions. The increased reducing ability of compound was directly proportional to the increased absorbance (700 nm) of the reaction solution. Results obtained were compared with ascorbic acid, which was taken as the gold standard [22].

Ferrous ion chelating potential of PGCMNPs was analyzed using the procedure of [23]. The reaction mixture contained different concentrations of PGCMNPs, ferrous chloride (2 mM) and ferrozine (5 mM). Decreased formation of ferrous ion-ferrozine complex in the presence of PGCMNPs was noted by measuring the absorbance at 562 nm.

The SOR inhibiting ability of PGCMNPs was analyzed by the method of [24]. The reaction solution contained different concentrations of PGCMNPs, riboflavin (2 µM), methionine (3 mM), EDTA (100 µM), sodium phosphate buffer (50 mM, pH 7.8) and nitro blue tetrazolium (75 µM). The formation of blue colored formazan was noted at 560 nm after 10 minutes of light exposure from a fluorescent lamp and compared with the control in the dark.

LPO inhibiting ability of PGCMNPs was determined by the method of [25]. The reaction mixture contained egg homogenate, PGCMNPs, ferrous sulfate (0.07 M), acetic acid (20% v/v, pH 3.5), thiobarbituric acid (0.8% w/v in sodium dodecyl sulfate) and butanol (99%, 5 mL). The absorbance of organic upper layer was read at 532 nm and compared with the control without treatment.

### Cellular uptake of PGCMNPs in human liver *cancer* cell line (HepG2)

In order to study the cellular uptake of PGCMNPs in HepG2 cells, PGCMNPs were tagged with FITC (fluorescein isothiocyanate) as described in an earlier study [18]. The successful synthesis of FITC tagged PGCMNPs (PGCMNPs-FITC) and characterization was provided in the supplementary data 2. For the cellular internalization studies, HepG2 cells (2×10^6^) were incubated with PGCMNPs-FITC (50 μg/mL) in the 6-well plate and incubated at 37 °C and 5% CO_2_ for 24 h. Cells were then measured using flow cytometer and data were analyzed using Cytoexpert software (Beckman Coulter life sciences, Indianapolis, United States).

### In vivo assessment of PGCMNPs

All the animal experiments were performed after the approval of institutional animal ethics committee (IAEC/KMC/85/2017) and study was conducted as per the guidelines of “committee for the control and supervision of experiments on animals (CCSEA)”. Male BALB/c mice (25-30 g, 6-8 weeks) were accommodated in the university’s central animal house facility. The animals from all the groups were provided with clean drinking water *ad libitum* and standard animal pellet diet (VRK Nutritional solutions, Maharashtra, India). All animals were acclimatized for 1 week before starting experiments.

#### Hemolysis assay

In order to determine the interaction of PGCMNPs with blood, prior to in vivo studies hemolysis assay was performed as per the method of [26] with minor modifications. Briefly, blood from the retro orbital plexus of the mice was collected in lithium heparin tubes and then centrifuged at 100⨯g for 5 minutes. Buffy coat was aspirated, RBCs were suspended in normal saline and incubated with different concentrations of PGCMNPs. The rate of hemolysis was determined after 3 hours of incubation and compared with triton-X as positive control. The results were expressed as Mean ± SD.

#### In vivo toxicity evaluation of PGCMNPs

For the toxicity evaluation of PGCMNPs, 30 mice were randomly divided into 3 groups (n=10 each). Test groups received PGCMNPs intravenously at a dosage of 5 mg/kg bw (low dose), and 10 mg/kg bw (high dose group). No special treatment was provided to the animals in the Control group. After 24 hours of dosing, 5 animals from each group were randomly euthanized. The remaining animals were monitored for 29 days for behavioral and physiological changes and on the 30^th^ day of the investigation, the remaining 5 animals from each group were euthanized. Liver, kidney, testis and spleen were analyzed histopathologically (H & E staining as per the standard laboratory procedure) for reticuloendothelial uptake and reproductive toxicity. Blood sera were also analyzed for liver injury markers (aspartate transaminase (AST), alanine transaminase (ALT) and alkaline phosphatase (ALP)) and kidney injury markers (urea, uric acid and creatinine) using standard kits (Agappe diagnostics Ltd., Kerala, India).

### Biodistribution studies of PGCMNPs

To determine the localization of PGCMNPs in various organs, in vivo biodistribution studies of PGCMNPs were performed. 30 mice were randomly divided into test and control groups, and biodistribution study was conducted at time intervals of 1 hour, 3 hours, and 24 hours respectively. Test group animals were administered with PGCMNPs (1.25 mg/kg bw) and Control group animals did not receive any special treatment. At each of the three time points, 5 animals each were sacrificed to harvest blood, heart, intestine, liver, spleen, brain, kidney, lungs, and testis. The iron distribution (ferric iron) of all the above organs was determined using Prussian blue staining [27]. Further, the concentration of cellular iron in each organ was estimated by Ferrozine assay [28].

### In vivo anticancer therapeutic potential of PGCMNPs

#### Development of HCC model and PGCMNPs treatment

In order to study in vivo anticancer therapeutic effect of PGCMNPs, mouse model of hepatocellular carcinoma was developed as described earlier [18, 29]. Briefly, *N*-*nitrosodiethylamine* (cumulative dose, 200 mg/kg bw) was injected intravenously to the mice (n=30) every week for 8 weeks. After 16 weeks of first dosing, the presence of HCC was confirmed by analyzing the levels of glypican-3 (GPC-3) in the blood serum of animals. The animals with a 2-fold increase in the GPC-3 levels as compared to the normal levels were randomly divided into TUMOR and TUMOR+PGCMNPs groups (n = 12 each). PGCMNPs and TUMOR+PGCMNPs group animals were administered with PGCMNPs (1.25 mg/kg bw) intravenously, once every week for the duration of 1 month. No special treatment was provided to Control and TUMOR group animals. At the end of study period, all the animals were euthanized to assess anticancer therapeutic potential of PGCMNPs in terms of tumor statistics, tumor dielectric properties, status of the antioxidants and cell death (schematic diagram of in vivo anticancer study was provided in the supplementary data 3).

#### Status of liver tumors

After completion of the treatment period, animals were sacrificed to note the presence of tumors. The tumors were categorized as big tumors (size ≥ 3 mm) and small tumors (< 3 mm) based on their visible size. The hepatosomatic index (HSI) of each animal was noted by determining the ratio of liver weight to the total body weight of the animals. Tumor multiplicity was determined as total tumor count of each animal to the total count of animals bearing tumors. Further, liver/ liver tumors were also assessed for the histopathological changes using H & E staining according to standard laboratory procedure.

#### Dielectric properties of hepatic tumors

In order to differentiate high and low grade tumors, electrical conductivity of liver tumors were measured using two-pin silver electrode as explained previously [30]. Prior to the measurements, probe was assessed for the errors and calibrated using NaCl (aq) solution. The conductance was measured at 8 different regions of liver using impedance analyzer (Hioki-IM3570, Japan) in the frequency range of 4 Hz-5 MHz. The tumors were categorized based on their electrical conductivity as percentage of tumors showing high degree of conductivity.

#### Assessment of ferroptosis

##### LPO and antioxidant enzyme systems

The liver of all the animals were homogenized using tris HCl buffer and divided into two parts. One part of the homogenate was used to determine LPO, and glutathione reduced (GSH). Other part of the homogenate was centrifuged (10,000 ⨯ g, 30 minutes) at 4 °C. The supernatant was then used for the estimation of glutathione reductase (GR), glutathione peroxidase (GP) and superoxide dismutase (SOD).

The extent of LPO in liver tumors was estimated by the method of [31]. The assay was based on the formation of malondialdehyde-thiobarbituric acid complex, which was measured at 535 nm. Results were presented as nmole of complex formed/ mg protein/minute.

GSH levels in the liver homogenate was determined as the amount of non-protein-sulfhydryl groups [32]. The analysis of GSH was based on the reduction of di-thio-nitro benzoic acid by the sulfhydryl groups of GSH into colored complex 2- nitro-5-mercaptobenzoic acid. The colored complex was then measured at 412 nm. Results were presented as nmole of GSH/ mg of protein.

The activity of GR was estimated according to the method of [33]. The GR present in the test sample catalyzes the reduction of glutathione in the presence of reducing agent NADPH. The amount of NADPH utilized during the reaction was indirectly measured and the activity of GR was expressed as nmole of NADPH used/minute/mg of protein.

The activity of GPx was noted according to [34]. The GPx in the sample catalyzed the conversion of H_2_O_2_ into H_2_O in the presence of NADPH. The amount of NADPH utilized was measured as decrease in the OD at 340 nm. The activity of GPx was presented as nmole of NADPH used/minute/ mg of protein.

The activity of SOD was determined by the method of [35]. The SOD in the sample catalyzed the conversion of superoxide radicals into H_2_O_2_ and O_2_. The activity of SOD capable of converting superoxide radicals produced during the oxidation of hydroxylamine hydrochloride was noted at 560 nm and presented as international units/mg of protein.

##### TUNEL assay

TUNEL assay was used to analyse the type of cell death in liver tumors. TUNEL assay was based on the addition of labelled de-oxy-uridine triphosphate (ie., biotinylated nucleotide conjugated to bromo-deoxy-uridine (Br-dUTPs)) to the 3’-OH ends of DNA fragments in the presence of enzyme terminal de-oxy-nucleotidyl transferase (TdT). In this assay, paraffinized tissue slides were dewaxed, rehydrated, stained as per the kit procedure and observed under the light microscope (Labomed, Lx-300, USA). The sections were analysed for the presence of TUNEL positive cells, and apoptotic index was expressed as percentage of apoptotic cells.

### Statistical analysis

Data were presented as Mean ± SD. Shapiro-Wilk test was used to analyze the normality of data and Levene’s test was used to analyze the homogeneity of variance respectively. Data were analyzed using one-way ANOVA. Further, Tukey’s test was used as post-hoc test for the comparison of groups for statistical significance. The student’s t-test was used to analyze the statistical significance of tumor multiplicity. P value ≤ 0.05 was considered to be statistically significant.

## Results

### Physical properties of PGCMNPs were suitable for in vivo anticancer applications

Physical characterizations of PGCMNPs such as solubility, stability, size, crystal structure and magnetic properties were very important in determining the in vivo behavior of PGCMNPs. In this study, PGCMNPs were brown in color, exhibited good solubility at neutral/physiological pH. PGCMNPs were also found to be stable at -20 °C up to 15 days. A detailed description of the solubility and stability of PGCMNPs were provided in the supplementary data 4 and 5.

**Fig. 1:**
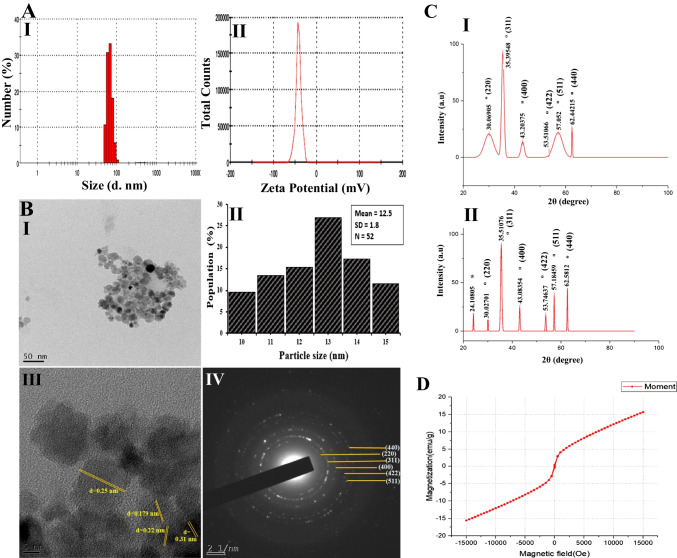
Size distribution, representative ζ -potential, high-resolution TEM images, X-ray diffraction patterns and vibrating sample magnetometry of PGCMNPs (**A**) **I.** Hydrodynamic size distribution of PGCMNPs (scale: 0–1000 nm), **II.** ζ-potential of PGCMNPs (Scale: -200–200 mV) (**B**) **I.** PGCMNPs were quasi-spherical in shape (scale: 50 nm) **II.** Mean particle size of PGCMNPs **III.** HR-TEM images of PGCMNPs with lattice fringes **IV.** SAED patterns of PGCMNPs (**C**) **I.** X-ray diffraction patterns of magnetite (Fe_3_O_4_) **II.** X-ray diffraction patterns of PGCMNPs. The peaks of PGCMNPs corresponding to the crystal planes of magnetite (Fe_3_O_4_) are marked as (°). The peak corresponding to PGC is marked as (*) (**D**) Magnetization curve for PGCMNPs displaying superparamagnetic properties. Remnant magnetization or remaining magnetization (M_r_) and coercivity (H_c_) was observed to be M_r_≈0.41 emu/g and H_c_≈63.3 Oe, respectively. The very negligible M_r_ and H_c_ values of PGCMNPs confirmed their superparamagnetic state

The hydrodynamic size of PGCMNPs was determined to be in the range of (50-105) nm with maximum number of particles of 60 nm (Fig. 1A.I). Similar findings were also noted when we performed nanoparticle tracking analysis (NTA) of PGCMNPs. Narrow range of size distribution was observed with maximum number of particles (90% of the particles) <100 nm (supplementary data 6). Further, the zeta potential of PGCMNPs was found to be -38.7 ± 3.1 mV which is considered to be a good enough potential to stop the aggregation of particles and oppose the Van der Waals forces (Fig. 1A.II). The polydispersity index (PDI) of PGCMNPs was found to be 0.39 ± 0.01, which suggested uniform size distribution of particles at physiological pH.

In order to determine the morphology, core size and crystal structure of PGCMNPs, the HR-TEM was performed. The HR-TEM micrographs were shown in the Fig. 1B.I-IV. PGCMNPs exhibited quasi-spherical shape and relatively uniform size distribution (Fig. 1B.I). The average core size of PGCMNPs was found to be 12.5 ± 1.8 nm (n=52) (Fig. 1B.II). PGCMNPs demonstrated crystalline nature as indicated by the presence of parallel lattice fringes and selected area electron diffraction (SAED) pattern for the phases of the crystal (Fig. 1B.III-IV). The crystalline properties and phases of PGCMNPs were further confirmed through X-ray powder diffraction (XRD) (Fig. 1C.I and 1C.II). The peaks for PGCMNPs were observed at 2θ of 30°, 35°, 43°, 53°, 57°, and 62° that corresponded to the crystal planes of (220), (311), (400), (422), (511), and (440), respectively. The observed diffraction patterns match with the characteristic diffraction pattern of the inverse spinel structure of Fe_3_O_4_. The peak positions of Fe_3_O_4_ in PGCMNPs remains unchanged as compared to magnetite (Fe_3_O_4_) (ICDD#75627) which demonstrated that binding of PGC did not result in the phase change of magnetite. However, PGCMNPs also displayed an additional peak around 2θ of 24.1° which indicated PGC peak with orthorhombic structure. Similar pattern was also observed for the chitosan by [36] and [37]. The average crystalline size and lattice constant (distance between the two atoms in the unit cell) were found to be 13.47 nm and 0.25 nm, respectively, which correlated with HR-TEM observations.

The magnetic properties pertaining to PGCMNPs were measured using vibrating sample magnetometry at 300 K. Figure 1D shows a typical magnetization curve for PGCMNPs. The saturation magnetism (Ms) was found to be 15.63 emu/g which was lessor than the Ms of bulk magnetite (≈ 80 emu/g) reported in the literature [38, 39]. The existence of PGC on the surface of Fe_3_O_4_ reduced Ms due to quenching of surface moments. Furthermore, the retentivity or remaining magnetization (Mr) and coercivity (Hc) was observed to be Mr ≈ 0.41 emu/g and Hc ≈ 63.3 Oe, respectively. The negligible Mr and Hc values of PGCMNPs confirmed their superparamagnetic state.

Further, to determine the purity of the final compound (PGCMNPs) and the absence of precursor compound (PGC), TLC was performed (supplementary data 7). PGC presented big purple spot and PGCMNPs presented small purple spot at different positions on the plate. This indicated that PGC was successfully grafted onto PGCMNPs, with no detectable precursor impurity.

### PGCMNPs revealed the presence of key elements and chemical bonds

**Fig. 2:**
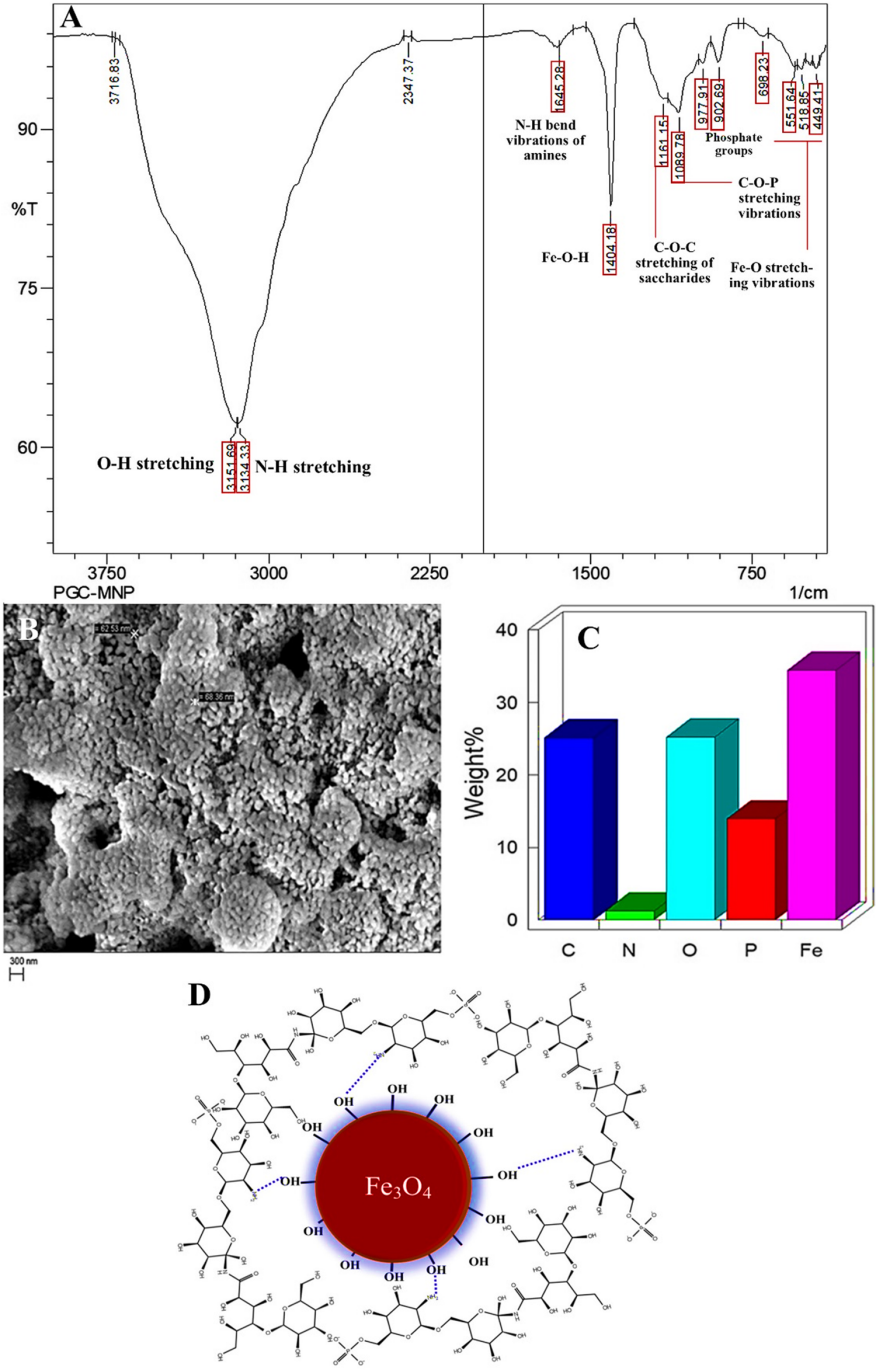
FT-IR spectrum, surface morphology and elemental composition of PGCMNPs. **A.** FT-IR spectrum of PGCMNPs **B.** Scanning electron micrograph of PGCMNPs with spherical morphology **C.** The elements in PGCMNPs were identified to be carbon (24.67 ± 4.37%), nitrogen (1.33 ± 0.09%), oxygen (23.36 ± 1.88%), phosphorous (11.97 ± 1.97%) and iron (38.37 ± 4.37%) **D.** Probable structure of PGCMNPs

The main elements detected in PGCMNPs were iron (38.37 ± 4.37%), carbon (24.67 ± 4.37%), nitrogen (1.335 ± 0.09%), oxygen (23.36 ± 1.88%) and phosphorous (11.97 ± 1.97%). Further, the bonding properties of PGCMNPs were determined by FT-IR spectroscopy. In the FT-IR spectrum of PGCMNPs, peaks corresponding to O-H stretching (3151 cm^-1^), N-H stretching (3134 cm^-1^), N-H bends (1645 cm^-1^), C-O-P stretching (1089 cm^-1^), phosphate groups (977 cm^-1^ , 902 cm^-1^), antisymmetric C-O-C stretching vibrations of saccharides (1161 cm^-1^), Fe-O stretching vibrations (698 cm^-1^ , 551 cm^-1^, 449 cm^-1^) and Fe-O-H vibrations (1404 cm^-1^) were observed. Overall, these results suggested the occurrence of ionic interaction between positively charged NH_3_^+^ groups of PGC and negatively charged OH^-^ of iron oxide core (Fig. 2A-C). The probable structure of PGCMNPs is provided in the Figure 2D.

### In vitro antioxidant properties of PGCMNPs

**Fig. 3:**
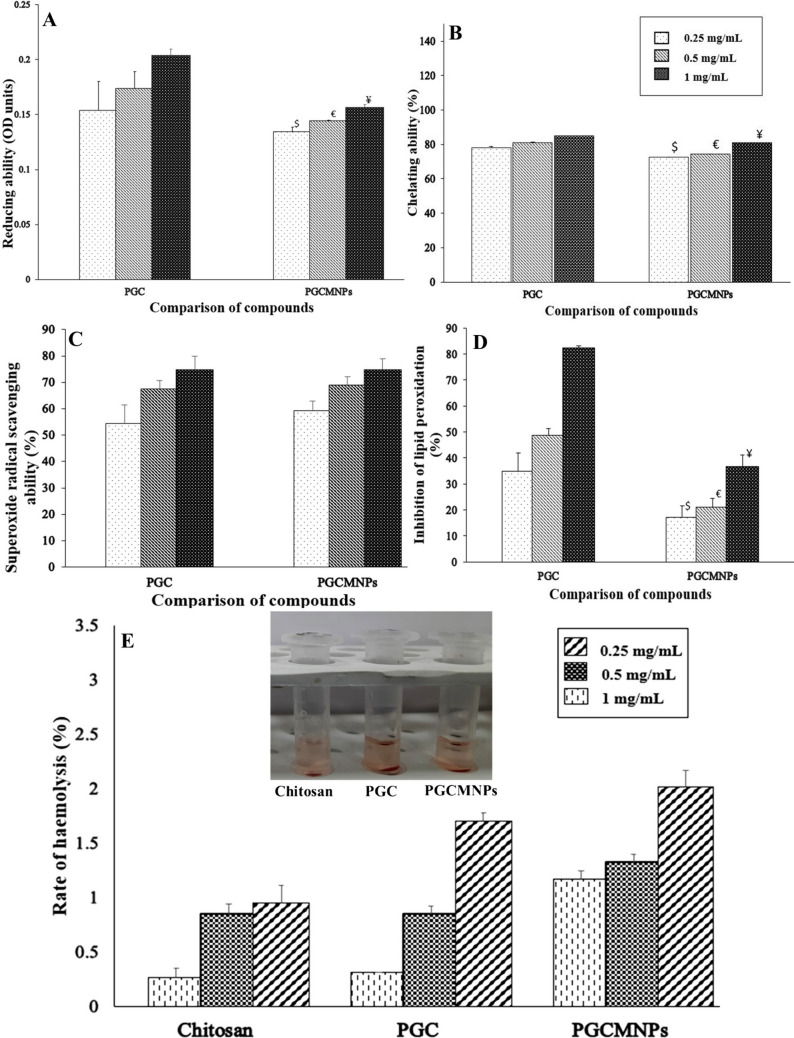
In vitro antioxidant properties and hemolytic activity of PGCMNPs. **A** Ferric ion reducing power of PGCMNPs when compared with PGC **B** Ferrous ion chelating ability of PGCMNPs when compared with PGC **C** SOR scavenging activity of PGCMNPs when compared with PGC **D** Lipid peroxidation scavenging ability of PGCMNPs when compared with PGC. Results presented as mean ± SD (n = 6). ^$^, ^€^ and.^¥^ signifies p ≤ 0.05 when compared with 0.25, 0.5, and 1 mg/mL of PGC, respectively **E** Hemolysis induced by PGCMNPs was < 5%, which was well within the acceptance criteria of ASTM E2524-08 standard. Results expressed as Mean ± SD (n = 3)

The ferric ion reducing capacity of PGCMNPs was significantly less as compared to the precursor compound PGC and gold standard ascorbic acid (p ≤ 0.05) at all tested concentrations (0.25 mg/mL, 0.5 mg/mL and 1 mg/mL) (Fig. 3A).

The metal chelating ability of PGCMNPs was determined to be 72% as compared to 78% at 0.25 mg/mL, 74% as compared to 80% at 0.5 mg/mL and 81% as compared to 84% at 1 mg/mL of PGC. A significant (p ≤ 0.05) decrease in the metal chelating ability of PGCMNPs was noted as compared to PGC and gold standard EDTA at all tested concentrations (Fig. 3B).

The superoxide scavenging activity was retained in the case of PGCMNPs, even after conjugation of PGC with magnetic nanoparticles. No significant (p ≤ 0.05) difference in superoxide scavenging activity of PGCMNPs was noted as compared to PGC at all tested concentrations (0.25 mg/mL, 0.5 mg/mL and 1 mg/mL) (Fig. 3C).

The inhibition of lipid peroxidation by PGCMNPs was significantly decreased at all the concentrations as compared to PGC (Fig. 3D). The inhibition of lipid peroxidation of PGCMNPs was determined to be 17.09% as compared to 37% at 0.25 mg/mL, 21% as compared to 48.6% at 0.5 mg/mL and 36.5% as compared to 82.24% at 1 mg/mL of PGC.

### PGCMNPs displayed no hemolytic activity and toxicity at tested parameters

The extent of hemolysis induced by PGCMNPs in RBC suspension over 3 hours of exposure at 37 °C was well within the acceptable range of 5% (ASTM E2524) [40] (Fig. 3E). The percentage hemolysis observed in PGCMNPs samples were 1.169 ± 0.075%, 1.329 ± 0.075%, and 2.02 ± 0.15% at the concentration 0.25, 0.5 mg/mL and 1 mg/mL respectively, which indicated that it is safe for in vivo administration.

**Fig. 4:**
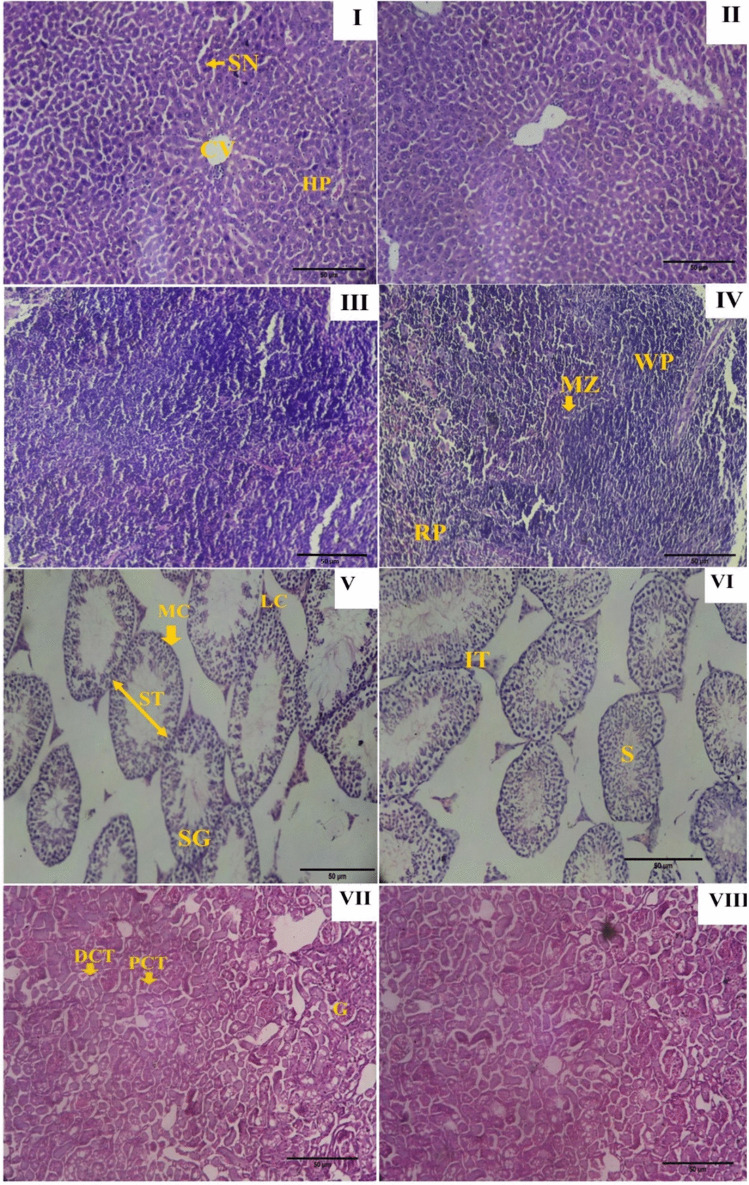
H & E-stained sections of animals treated with PGCMNPs. **I**, **II**: Microphotographs of liver displaying normal arrangement of hepatocytes (HP) with no condensation and regeneration of cytoplasm. Central vein (CV) and sinusoids (SN) were normal in appearance and RBC pooling was not observed. **III, IV:** Microphotographs of spleen were normal with distinguishable white pulp (WP), red pulp (RP) and marginal zones (MZ). **V,VI:** Microphotographs of testis displaying normal appearance of seminiferous tubules (ST) with sperm (S), spermatogonia cells (SG) bordered with peritubular myoid cells (MC) and groups of Leydig cells (LC). **VII,VIII:** Microphotographs of kidney with regular architecture displaying glomerulus (G), proximally convoluted tubule (PCT) and distally convoluted tubule (DCT)

PGCMNPs showed no toxic effects at 24 hours and 30 days after administration as indicated by the liver and kidney injury markers (Table 1). The histopathological studies also displayed no toxicity at tested doses (Fig. 4).

**Table 1:** In vivo toxicity evaluation of PGCMNPs **A**. Liver injury markers: Activities of AST, ALT and ALP post 24 h and 30 days administration of PGCMNPs at the dose of 5 mg/kg bw and 10 mg/kg bw. **B**. Kidney injury markers: Levels of urea, uric acid and creatinine post 24 h and 30 days administration of PGCMNPs at the dose of 5 mg/ kg bw and 10 mg/kg bw. Data were presented as mean ± SD and analysed using one-way ANOVA followed by post hoc test (Tukey’s test). P ≤ 0.05 was considered statistically significant

Time period	Group	A. Liver function tests (U/L)			B. Kidney function tests (mg/dL)		
		AST	ALT	ALP	Urea	Uric acid	Creatinine
24 h	Control	80.86 ± 3.88	71.992 ± 3.48	76.13 ± 4.22	28.28 ± 6.47	5.87 ± 1.51	0.47 ± 0.09
	5 mg/kg bw	82.86 ± 3.66	75.48 ± 5.24	77.14 ± 4.25	27.75 ± 4.10	5.99 ± 0.64	0.44 ± 0.03
	10 mg/kg bw	81.77 ± 3.27	72.89 ± 6.43	77.78 ± 4.84	30.12 ± 5.21	6.08 ± 0.77	0.43 ± 0.05
30 days	Control	110.17 ± 5.92	61.99 ± 4.08	167.74 ± 10.78	24.38 ± 3.24	13.26 ± 0.95	0.27 ± 0.00
	5 mg/kg bw	107.39 ± 13.10	65.48 ± 7.66	167.42 ± 11.29	26.97 ± 2.88	13.16 ± 0.95	0.26 ± 0.02
	10 mg/kg bw	105.78 ± 10.40	66.89 ± 5.84	160.11 ± 8.78	26.4 ± 5.61	13.82 ± 1.3	0.27 ± 0.00

### PGCMNPs were effectively internalized by liver cells

**Fig. 5:**
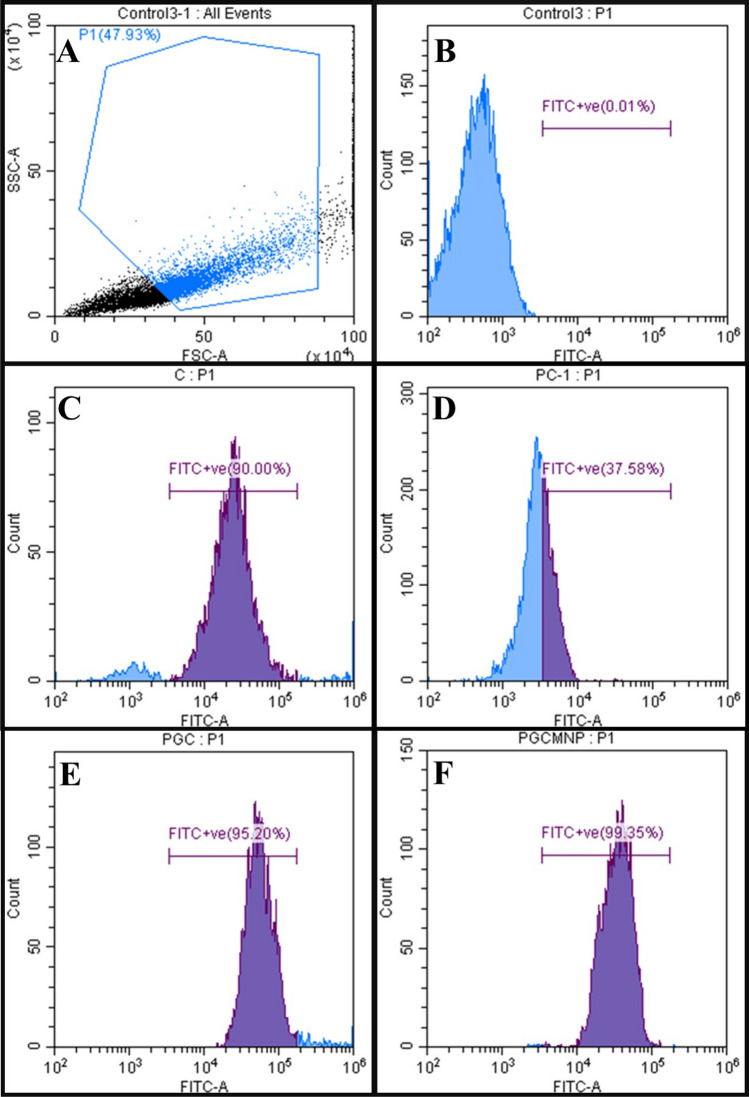
Flow cytometry images of PGCMNPs uptake: (**A**) Gating strategies for the experiment (**B**) Control HepG2 cells without PGCMNPs treatment (blue color is represented as HepG2 cells with no fluorescence). Representative results of HepG2 cells treated with (**C**) FITC-Chitosan (**D**) FITC-Phosphorylated Chitosan (**E**) FITC-PGC (**F**) FITC-PGCMNPs for 24 h (Blue color is presented as cells with no fluorescence; purple color is presented as cells with fluorescence)

**Fig. 6:**
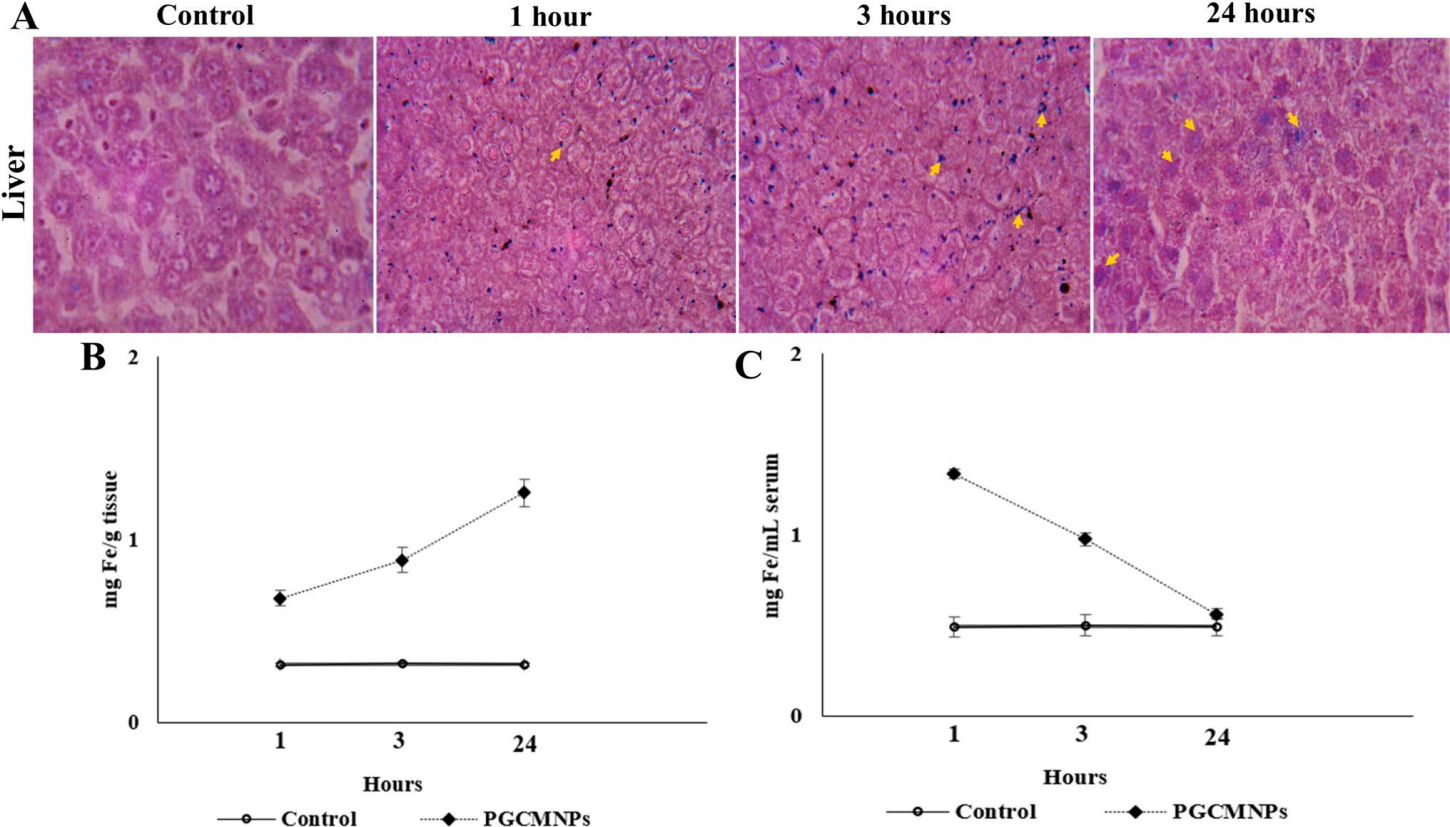
Biodistribution of PGCMNPs in the liver and blood serum. **A** Representative photomicrographs of liver stained with Prussian blue to detect iron deposits at 1 h, 3 h and 24 h after the intravenous administration of PGCMNPs (400X) **B** Changes in the Fe levels of liver at 1 h, 3 h and 24 h after the intravenous injection of PGCMNPs **C** Changes in the Fe levels of blood serum at 1 h, 3 h and 24 h after the intravenous injection of PGCMNPs. Data were presented as Mean ± SD

Cellular internalization study of PGCMNPs in HepG2 cells was performed to compare the uptake of PGCMNPs with precursor compounds; phosphorylated galactosylated chitosan (PGC), chitosan (C) and phosphorylated chitosan (PC). After 24 hours of incubation, highest uptake was noted in PGCMNPs group with the fluorescent intensity in the range of (99-100) % as compared to PGC group (95.16-98.76) % (Fig. 5E and F). The other precursor compounds chitosan and phosphorylated chitosan showed an uptake in the range (90-93.78) % and (37.58-39.74) % respectively (Fig. 5C and D). These results were further supported by in vivo biodistribution studies of PGCMNPs. Increased iron content was observed in the liver of PGCMNPs-injected animals compared to control animals. 1 hour post PGCMNPs administration, majority of the iron was observed in the liver sinusoids (about 114% increase was noted as compared to control). Further, at 3 hours 174% increase was noted which was increased to 296 % after 24 hours. Prussian blue staining after 24 hours of administration showed increase in the iron deposits within hepatocytes suggesting the internalization of PGCMNPs (Fig. 6). Corresponding decrease in the levels of iron in the blood serum was noted from 1 to 24 hours-post PGCMNPs administration. The other tissues such as kidney, lung, brain, spleen, intestine, heart, brain and testis had no obvious increased iron content at each time point after the administration of PGCMNPs (supplementary data 8). These results indicated that liver was the major target organ for PGCMNPs uptake.

### PGCMNPs demonstrated strong anticancer therapeutic potential against HCC

**Fig. 7:**
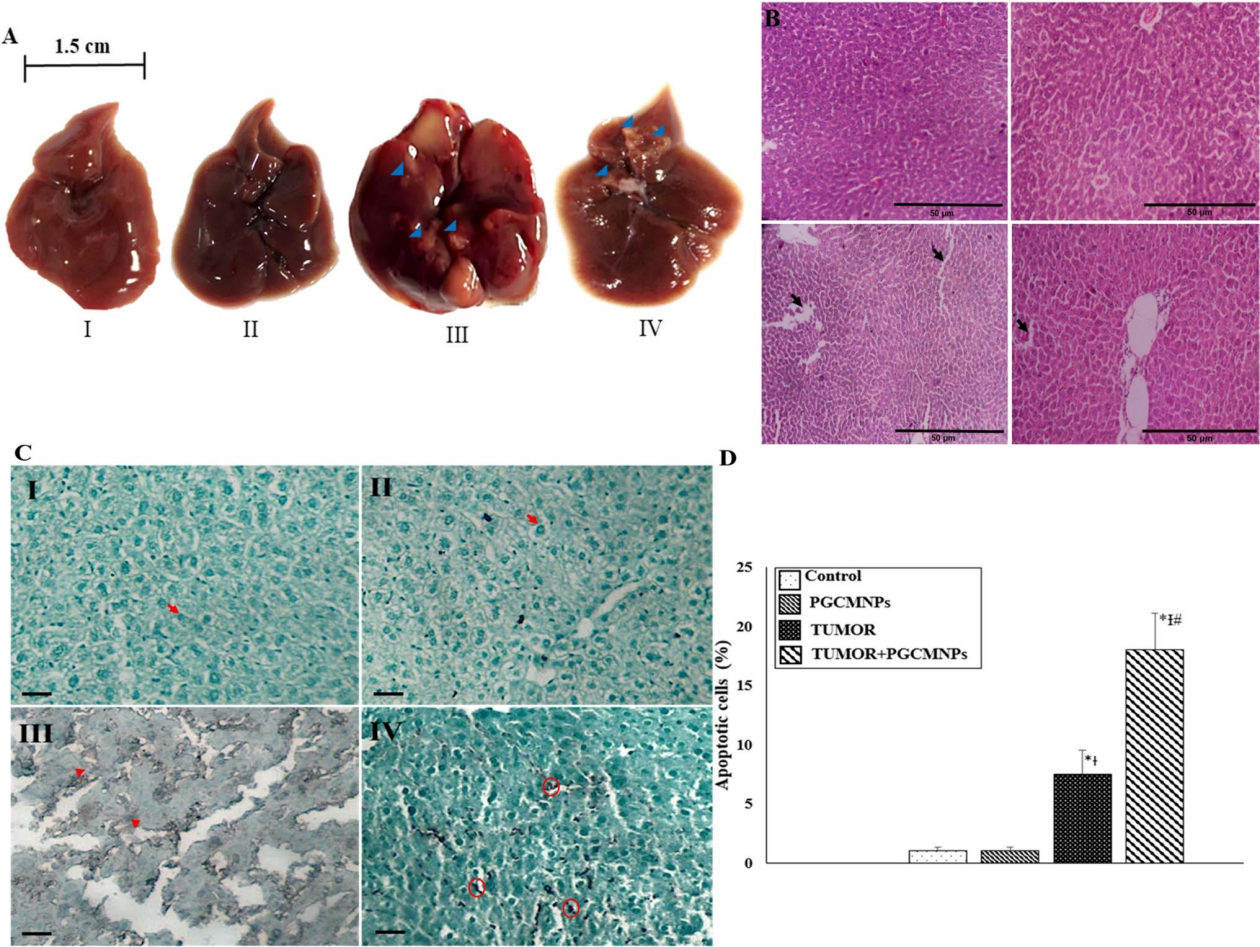
In vivo anticancer therapeutic potential of PGCMNPs: (**A**) Liver morphology after 4 weeks of PGCMNPs therapy. **(I, II)** Control and PGCMNPs groups displaying normal and distinct lobes **(III)** Liver in the TUMOR group displaying enlarged lobes with HCC nodules ≥ 3 mm (arrowhead) **(IV)** TUMOR + PGCMNPs group displaying abnormal liver with small tumors (arrowhead) (**B**) **H & E-stained sections of liver (I, II)** Histopathology of normal Control and PGCMNPs groups displaying normal arrangement of hepatocytes **(III)** Histopathology of TUMOR group displaying moderately differentiated HCC **(IV)** Histopathology of TUMOR + PGCMNPs group displaying well-differentiated tumor (magnification 100X). (**C & D**) **TUNEL assay in liver/liver tumors.** (**C**) **(I and II)** Photomicrographs of Control and PGCMNPs groups showing green stained nucleus in non-apoptotic cells (Arow) **(III)** Photomicrographs of TUMOR group showing brown-colored stains in nucleus and cytoplasm representing necrotic cells (arrow head) **(IV)** Photomicrograph of TUMOR + PGCMNPs group showing brown colored nucleus in cells undergoing apoptosis (circle) (magnification: 100X, scale bar: 50 µM) (**D**) Percentage of apoptotic cells in liver/liver tumors after 4 weeks of PGCMNPs treatment in TUMOR group animals (Data were expressed as mean ± SD and analysed using one-way ANOVA followed by post hoc test (Tukey’s test). *: represents (p ≤ 0.05) when compared with the Control group; ϯ: represents (p ≤ 0.05) when compared with PGCMNPs group; #: represents (p ≤ 0.05) when compared with TUMOR group)

The anticancer therapeutic potential of PGCMNPs was determined in NDEA-induced HCC model of mice. PGCMNPs therapy was initiated after confirming the presence of tumor via blood serum marker (GPC-3). However, PGCMNPs displayed good anticancer therapeutic effect on liver tumors which was confirmed from gross morphology of liver. The four weeks of PGCMNPs therapy evidently reduced the number of tumors in TUMOR+PGCMNPs group (total number of tumors: 7, 6 small tumors and 1 big tumor) as compared to TUMOR group (total number of tumors: 42, 28 small tumors and 6 big tumors). Additionally, the histopathology of tumors also confirmed well to moderately differentiated HCC in PGCMNPs treated TUMOR group (Fig. 7). In addition to this, TUMOR+PGCMNPs group had a greater number of animals without tumors (41.6%) when compared with TUMOR group without treatment (100%). PGCMNPs treatment also displayed significantly decreased (p ≤ 0.05) hepatosomatic index as compared to TUMOR group. The anticancer effect of PGCMNPs was also reflected in tumor multiplicity wherein, PGCMNPs treatment significantly (p ≤ 0.05) decreased the tumor multiplicity when compared to TUMOR group. Further, the electrical conductivity of tumors in the PGCMNPs treated TUMOR group (14.28%) was lower than tumors in the untreated TUMOR group (40.47%) (Table 2) which reflected that tumors in the treatment group were less aggressive compared to the untreated group.

**Table 2:** Tumor statistics, after 4 weeks of PGCMNPs treatment

Parameters	Control	PGCMNPs	TUMOR	TUMOR + PGCMNPs
Hepatosomatic index (HSI)	0.039 ± 0.004	0.0427 ± 0.0028	0.056 ± 0.005*	0.042 ± 0.001^ϯ^
Big tumors (≥ 3 mm)	-	-	14	1
Small tumors (< 3 mm)	-	-	28	6
Percentage of animals having tumors after treatment	-	-	100%	41.66%
Tumor multiplicity	-	-	0.722 ± 0.264	0.3125 ± 0.025^ϯ^
Tumor showing high degree of conductivity (%)	-	-	40.47%	14.28%

HSI was analysed using one-way ANOVA followed by post hoc test (Tukey’s test). Student's t-test was used to the statistical significance of tumor multiplicity

*: represents p ≤ 0.05 when compared with the Control group; ^ϯ^: represents p ≤ 0.05 when group is compared with TUMOR group.

### PGCMNPs treatment led to ferroptosis

PGCMNPs treatment to the NDEA challenged animals increased the apoptosis mediated cell death in the liver tumors, which was indicated by the dark brown stained nuclei. However, in the case of untreated TUMOR group, the brown stain was noticed both in the cytoplasm as well as in the nucleus which was indicative of necrotic cell death (Fig. 7C). The percentage of apoptotic index were calculated, and significantly higher (p ≤ 0.05) apoptotic index were observed in TUMOR+PGCMNPs group (18%) as compared to the TUMOR group (7.5%) (Fig. 7D).

A significant decrease (p ≤ 0.05) in the levels of GSH and antioxidant enzymes was noted in the TUMOR and TUMOR+PGCMNPs groups. However, significant (p ≤ 0.05) increase was noted in the PGCMNPs treated NDEA challenged animals compared to untreated animals. Additionally, a significantly increased cytoplasmic LPO was observed in TUMOR and TUMOR+PGCMNPs groups compared to control animals. A significant decrease in the cytoplasmic LPO was noted in the TUMOR group treated with PGCMNPs compared to untreated group (Table 3).

**Table 3:** Status of antioxidant defense system in liver/liver tumors after 4 weeks of PGCMNPs treatment

Parameters	Control	PGCMNPs	TUMOR	TUMOR + PGCMNPs
LPO (nmole/min/mg protein)	0.200 ± 0.020	0.221 ± 0.030	0.885 ± 0.061*	0.661 ± 0.067*^ϯ^
GSH (nmole/mg protein)	2.591 ± 0.293	2.306 ± 0.158	1.040 ± 0.055*	2.077 ± 0.062*^ϯ^
GSH-R (nmole/min/mg protein)	1.252 ± 0.041	1.309 ± 0.113	0.820 ± 0.026*	0.954 ± 0.051*^ϯ^
GSH-Px (nmole/min/mg protein)	1.485 ± 0.107	1.479 ± 0.058	0.789 ± 0.068*	1.109 ± 0.07*^ϯ^
SOD (IU/mg protein)	0.206 ± 0.010	0.201 ± 0.007	0.117 ± 0.019*	0.167 ± 0.01*^ϯ^

(Data were expressed as mean ± SD and analysed using one-way ANOVA followed by post hoc test (Tukey’s test). *: represents p ≤ 0.05 when compared with the Control group; ϯ: represents p≤0.05 when compared with TUMOR group).

## Discussion

HCC is one of the deadliest cancers [41]. The current management strategies for HCC are liver transplantation and surgical resection, percutaneous ethanol injection, microwave ablation, percutaneous radiofrequency ablation, cryoablation, irreversible electroporation, trans arterial chemoembolism (TACE) and trans arterial radio embolism (TARE) [42–44]. Most of these procedures are invasive and requires direct injection of treatment drug into the tumor tissue [45]. This can lead to hemorrhage, portal vein thrombosis, duct injury, diaphragmatic injury or liver abscess etc. [46, 47]. The targeted cancer therapies for HCC usually employ receptors like asialoglycoprotein receptor (ASGPR) [48], glycyrrhetinic acid receptor [49], transferrin receptor [50], somatostatin receptor [51], folate receptor [52], retinoic acid receptors [53] or epidermal growth factor receptor [54] etc. to deliver drugs to liver tumors. Amongst them, ASGPR is the most explored or common target receptor and employed widely by different carriers for active drug delivery to hepatic tumors cells [48, 55, 56]. In this study, we have used our earlier developed PGC as coating material for SPIONs (PGCMNPs) to utilize the benefits of PGC and SPIONs in terms of both active and passive tumor targeting of liver tumors. The uptake of these nanoparticles into the cell is determined by various physical and chemical properties like size, shape, charge, composition and surface coating [2]. These characteristic parameters further determine the membranal interaction and its subsequent entry into the cell [57]. Therefore, to increase the uptake, PGCMNPs were optimized for all the physical and chemical properties [20]. When in the blood, nanoparticles interact with blood components which consists of cells, numerous proteins and water. This interaction is important as it will decide the fate of nanoparticles [58]. The presence of negative charge on the surface of nanoparticles protect them from being engulfed by macrophages as well as reduces the aggregation of particles [18]. The surface coating or the functional layer of the nanoparticle determines the stability and targeting ability in an in vivo system. Bare nanoparticles once suspended in the biological fluids like blood, will largely interact with proteins forming “protein corona” [59]. The irreversible attachment of these proteins onto the surface of nanoparticles can severely alter their behaviour in vivo. Francia and group explored the effects of protein corona on the silica nanoparticles which were targeted towards the low-density lipoprotein receptors and observed less uptake of nanoparticles in the presence of higher amount of serum proteins [60]. Protein molecules attached to the nanoparticles can also block the ligands and can interfere with receptor mediated-uptake, thus increasing its retention in the blood. Therefore, to prevent the blocking of ligands, polymers such as polyethylene glycol or chitosan are usually coated onto the surface of the nanoparticles. Additionally, nanoparticles without surface coating can also undergo rapid agglomeration and can be easily taken up by the macrophages [61]. Therefore, to prevent this process, the surface of the nanoparticles is covered with polymers [62]. Additionally, size of the particles determines their entry into the tumor vasculature [63]. The tumor blood vessels have pore size bigger than blood vessel of a normal tissue which make them leaky, and nanoparticles of appropriate size can easily pass through them [64]. This type of accumulation of nanoparticles within the tumor tissue is called “EPR effect” [65]. The mean core size and hydrodynamic size of PGCMNPs was determined to be 12.5 nm and 60 nm respectively which was appropriate for showing EPR effect. The size was also found to be ideal for interaction and subsequent uptake via ASGP receptors [66]. This was supported by both in vitro cellular uptake studies and in vivo biodistribution studies. In in vitro cellular uptake study compared to the uptake of coating material PGC (95.16-99.14%), higher uptake of PGCMNPs (99-100%) was noted in hepatoma cells over a period of 24 hours. The higher uptake in the case of PGCMNPs might also be attributed to the increased surface area of the ligands as compared to PGC. The smaller sized spherical particles provide larger surface area for the attachment of the ligands. Increase in ligand density all around the particle thereby increase the affinity towards receptor for the total engulfment via receptor mediated endocytosis [67]. Further, 1 hour, 3 hours and 24 hours in vivo biodistribution studies of PGCMNPs suggested time-based uptake of PGCMNPs in the liver. The complete internalization of PGC coated iron oxide nanoparticles inside the hepatocytes was clearly observed after 24 hours of administration, thereby suggesting increased stability and localization in the liver.

The coating of PGC onto the iron oxide nanoparticles was validated by FT-IR spectroscopy. The ionic interactions were noted between positively charged amino groups (NH_3_^+^) of PGC and negatively charged groups (-OH^-^) of iron oxide. In the FT-IR spectrum of PGCMNPs, the presence of peak at 1645 cm^-1^ assigned to N-H bend vibrations, peak at 1089 cm^-1^ assigned to C-O-P stretching vibrations along with the peaks pertaining to the iron oxide core (Fe-O) at 551 cm^-1^ proved that PGC was successfully coated onto iron oxide nanoparticles.

Another major advantage of using SPIONs as carriers is that they can be used as multifunctional therapies where they can be targeted to tumor sites with external magnetic field and then once located can be employed for magnetic hyperthermia tumor ablation. Hyperthermia has been proposed as an anticancer treatment, wherein under an applied magnetic field the SPIONs generate heat due to relaxation losses. The raise in temperature from 40-45 ºC further lead to cell death of temperature sensitive cancer cells without damaging the normal cells [68]. Magnetic targeting and hyperthermia causing abilities of SPIONs are greatly influenced by the type of iron oxide ore and its crystal structure [69]. Iron oxide is a compound which presents more than one crystalline structure each with unique physical properties [70]. The three main crystalline forms of iron oxides are hematite (α-Fe_2_O_3_), magnetite (Fe_3_O_4_) and maghemite (γ-Fe_2_O_3_) [71]. Amongst them, the nanoparticles prepared from magnetite (Fe_3_O_4_) have received significant considerations in the biomedical applications due to their outstanding characteristics including biocompatibility, desirable surface chemistry, narrow particle size distribution (<100 nm) and superparamagnetism [72].

The XRD analysis of PGCMNPs confirmed the peaks pertaining to all the six phases of magnetite core representing the cubic inverse spinel crystalline structure. The PGC in the outer shell of the magnetite was also represented by an additional peak pertaining to the orthorhombic structures of the saccharides. The presence of magnetite was also confirmed by well-separated quasi-spherical nanoparticles and SAED patterns observed in the TEM micrographs. Interatomic distances (or the distance between the parallel lattice fringes) was found to be 2.5 Å from the (311) phase of the Fe_3_O_4_ crystal which was in concordance with the (311) planes of inverse spinel Fe_3_O_4_ nanoparticles [73], thereby, validating the presence of magnetite in PGCMNPs. The validation of crystal structure of PGCMNPs and size were important to determine its superparamagnetic property. “Superparamagnetic iron oxide nanoparticles” (SPIONS) is of great importance in the field of targeted drug delivery since they easily get concentrated in the tumor site under the influence of external magnet and also represent no remanence magnetism [74]. The absence of remnant magnetism is extremely important in in vivo applications as zero or negligible remanence guarantees homogenous dispersion of the nanoparticles and reduces field related aggregation of nanoparticles. This in turn ensures decrease in macrophage-mediated phagocytosis and nanoparticles aggregation related blockages or thrombosis of the capillaries [75]. VSM analysis of PGCMNPs revealed no hysteresis, no remnant magnetization and negligible coercivity thus displaying the necessary conditions for superparamagnetic behaviour. Overall, the presence of these unique properties in PGCMNPs endow them with the ability to respond to the external magnetic field and can be further used for hyperthermia/theranostic/multifunctional cancer treatment applications. Therefore, PGCMNPs with these favourable properties were further investigated for their in vivo anticancer therapeutic ability.

Before the investigation of anticancer therapeutic potential, PGCMNPs were assessed for hemocompatibility and in vivo toxicity. PGCMNPs displayed <5% hemolysis and hence was suitable for intravenous administration (ASTM standard E2524-08) [40]. They were also suitable for in vivo administration as demonstrated by short and long-term toxicity studies. The anticancer therapeutic potential of PGCMNPs against HCC was confirmed in *N- nitrosodiethylamine*-induced HCC mice model. Hepatic cancer model was developed using *N-nitrosodiethylamine* (NDEA) as carcinogen. The development of tumors in animals was confirmed using serum marker glypican-3 (GPC-3). GPC-3 is an excellent serum marker that appears quite early in the development of hepatic cancer [76]. The animals with 2-fold higher levels of GPC-3 as compared to normal animals were taken as HCC positive. HCC bearing animals were randomly divided into Tumor and TUMOR+PGCMNPs groups. The TUMOR+PGCMNPs group animals were treated with PGCMNPs (1.25 mg/kg bw) for one month. After one month of treatment, the evident decrease in the number of tumors and tumor multiplicity in PGCMNPs treated NDEA challenged animals markedly revealed its anticancer therapeutic potential against HCC. These findings were also substantiated by histopathological analysis of liver tumor tissues. The tumors obtained in the untreated group showed the presence of undifferentiated HCC whereas, lower grade tumors were observed in the treatment group. This was further supported by the electrical measurements of these tumors. The electrical conductivity of hepatic tumors is positively correlated with the histopathological grade of hepatic tumors [30]. The advanced stage hepatic tumors demonstrate a very high electrical conductivity. This might be due to the presence of increased necrotic areas in advanced hepatic tumors. The increased necrotic areas allows more amount of electrical current to pass through it and thus demonstrates higher conductivity [77, 78]. The decrease in the presence of high conductivity tumors in treatment group when compared to untreated group clearly indicated good anticancer therapeutic potential of PGCMNPs against HCC. In our earlier study, we have reported anticancer activity of coating material “PGC” [18]. In comparison with PGC (tumor presence: 50% and tumor multiplicity: 0.385±0.144) improvement in anticancerous properties in terms of tumor statistics were noted in the case of PGCMNPs (tumor presence: 41% and tumor multiplicity: 0.312±0.025). Further, PGCMNPs treatment also decreased the percentage of tumors with high degree of conductivity (14.28%) as compared to PGC (16.66%). Overall, these results suggested improved anticancerous properties in PGCMNPs compared to PGC.

**Fig. 8:**
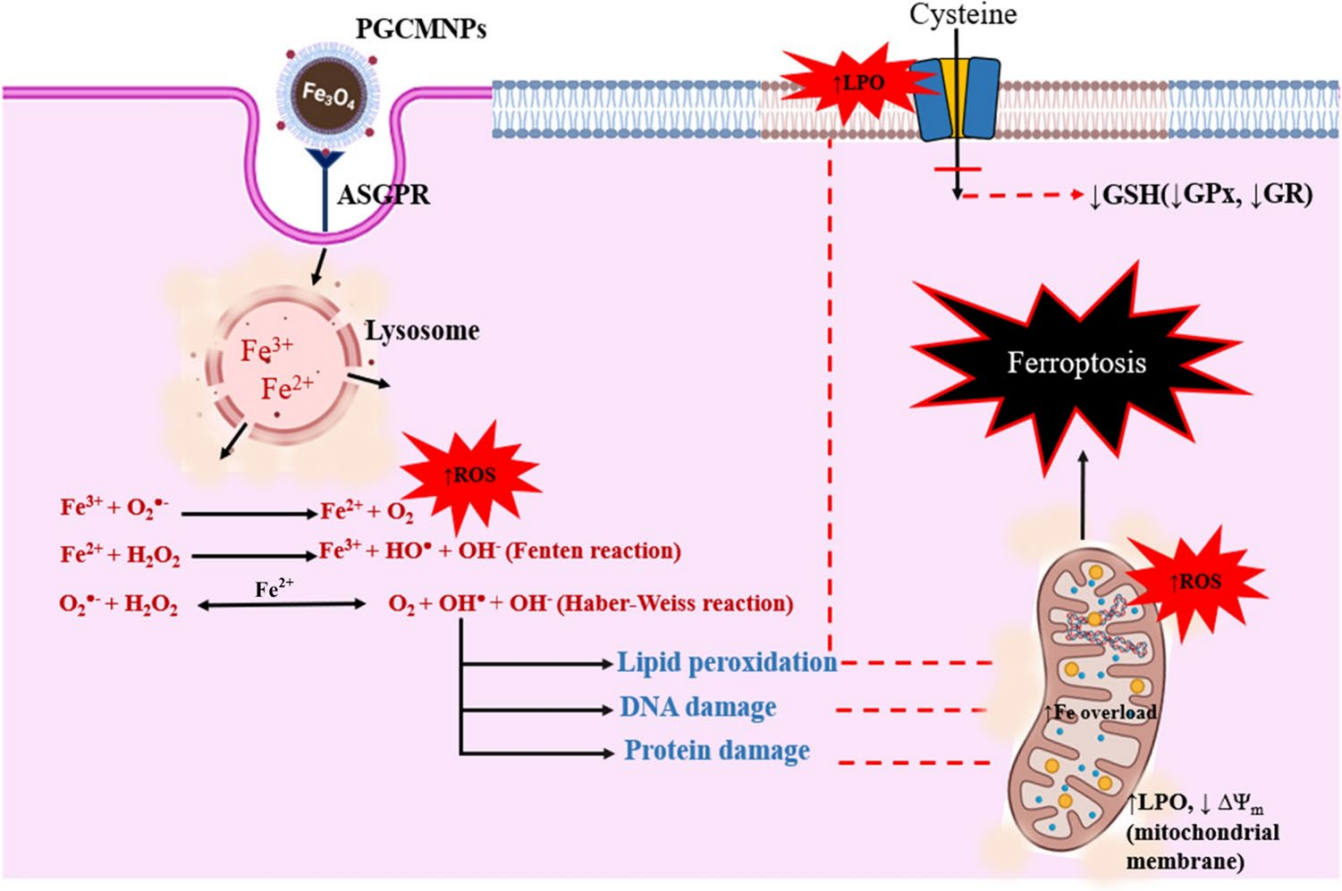
Mechanism of action of PGCMNPs. PGCMNPs were internalized through clathrin mediated endocytosis and further degraded into free ferrous and ferric ion in the lysosomes. The released iron gets accumulated in the cytosol and mitochondria and increase oxidative stress through Fenten and Haber- Weiss reaction. The oxidative stress further leads to peroxidation of polyunsaturated lipids in membrane and modifications of the transporters on the transporters on the membrane. The decreased uptake of cysteine for the formation of glutathione and increased lipid peroxidation leads to higher oxidative stress in mitochondria and ferroptosis

The anticancer activity of PGCMNPs noted in this study might be mediated through the process of iron-induced apoptosis or ferroptosis. Ferroptosis is a distinct type of controlled cell death described as an iron-mediated process of excessive peroxidation of lipids [79]. PGCMNPs once internalized dissolves in the acidic environment of the lysosomes and undergo degradation in which iron is released in the cytoplasm [80, 81]. The iron released in the cytoplasm leads to iron-mediated redox reactions called as Fenton’s reactions. During Fenton reaction, the hydrogen peroxide (H_2_O_2_) reacts with iron (II) (Fe^2+^) to form hydroxide (OH^−^) and hydroxyl radicals (OH^•^). The presence of Fe^2+^ in the cytoplasm also acts as catalyst for Haber-Weiss’s reaction, which is usually thermodynamically unfavorable in a biological system. This reaction further generates highly reactive radicals (OH^•^) [16]. The ROS generated can lead to peroxidation of polyunsaturated fatty acids of the biological membranes generating lipid peroxides (LPO) [82]. Alteration in the biological membranes, further damage the integrity and structure of organelles especially the mitochondria leading to decreased mitochondrial membrane potential, ATP depletion and eventually causing ferroptosis [83] (Fig. 8).

The maintenance of lipid bilayer homeostasis and prevention of LPO formation is achieved by an enzyme called glutathione peroxidase (GPX) [84]. However, several inducers or promoters that drive ferroptosis inactivates GPX directly or indirectly and increase the levels of LPO [84–86]. In this study, PGCMNPs presented decreased in vitro scavenging free radical scavenging activity compared to coating material PGC. Further, after a month of PGMNPs therapy, decrease in the levels of GSH (1.08 folds), GR (1.29 folds), GPx (1.25 folds) and increase in LPO (0.768 folds) was noted in the case of PGCMNPs treated tumors. Further, an increase in apoptotic cell death as demonstrated by TUNEL assay was also observed in PGCMNPs treated tumors suggesting a probable cause of cell death as ferroptosis.

In conclusion, PGCMNPs showed good anticancer therapeutic activity against HCC. This type of agent may also be a good candidate for theranostic applications [87–89] whereby both diagnosis and tumor treatment can be achieved using single agent. Further, study also reported synthesis method which can be used for synthesis of radioactive ^32^PGCMNPs. This agent can further offer multimodal cancer treatment options via radiation ablation, biochemical pathways, MRI-guided ferroptosis as well as magnetic hyperthermia.

## Supplementary Information

Below is the link to the electronic supplementary material.

Supplementary file1 (DOCX 10162 KB)

## Data Availability

The data generated during the study are available from the corresponding author on a reasonable request.
