# Supplementary material for: Anticancer therapeutic potential of multimodal targeting agent- “phosphorylated galactosylated chitosan coated magnetic nanoparticles” against N-nitrosodiethylamine-induced hepatocellular carcinoma
Source: Drug Deliv Transl Res. 2024 Jul 11;15(3):1023–42. doi: 10.1007/s13346-024-01655-1 (PMC11782354; doi:10.1007/s13346-024-01655-1)
Supplement: Supplementary file 1 — Supplementary file1 (DOCX 10162 KB) [file 13346_2024_1655_MOESM1_ESM.docx]

**Supplementary data 1. PGCMNPs synthesis apparatus**


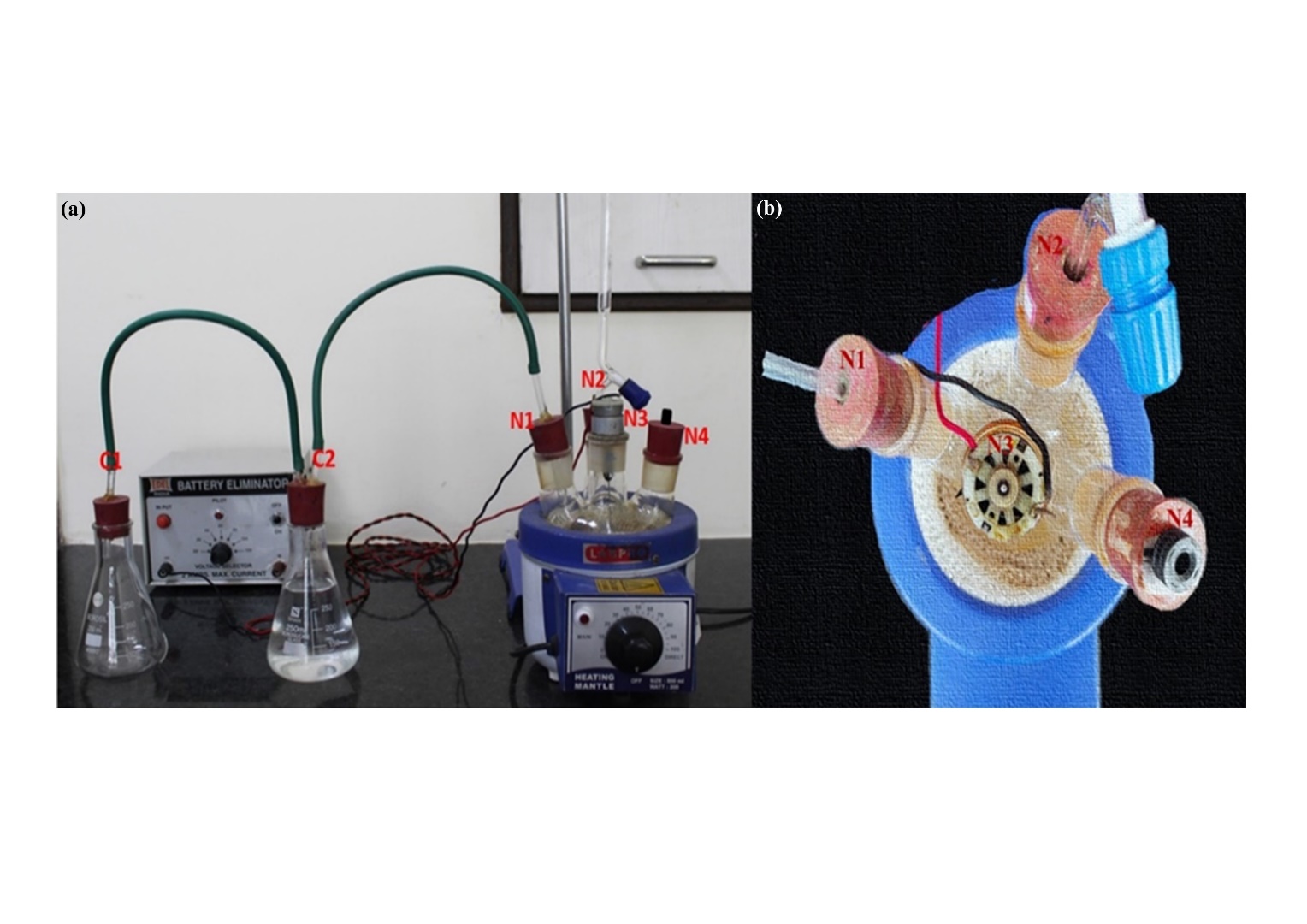
**Figure 1. Design of apparatus for PGCMNPs synthesis: (a)** Four-necked in situ PGCMNPs synthesizing apparatus: **C1:** Conical flask for the synthesis of N_2_ gas, **C2:** Conical flask with NaOH (0.1 N) for the dissolution of other gases, **N1:** Inlet for N_2_ gas, **N2:** Cork with burette for the dropwise addition of liquid ammonia, **N3:** Overhead stirrer, **N4:** Inlet for the addition of PGC solution **(b)** Top view of the four necked flask with inlets.

**Supplementary data 2. Cellular internalization of PGCMNPs**

**FT-IR characterization of FITC-tagged PGCMNPs**


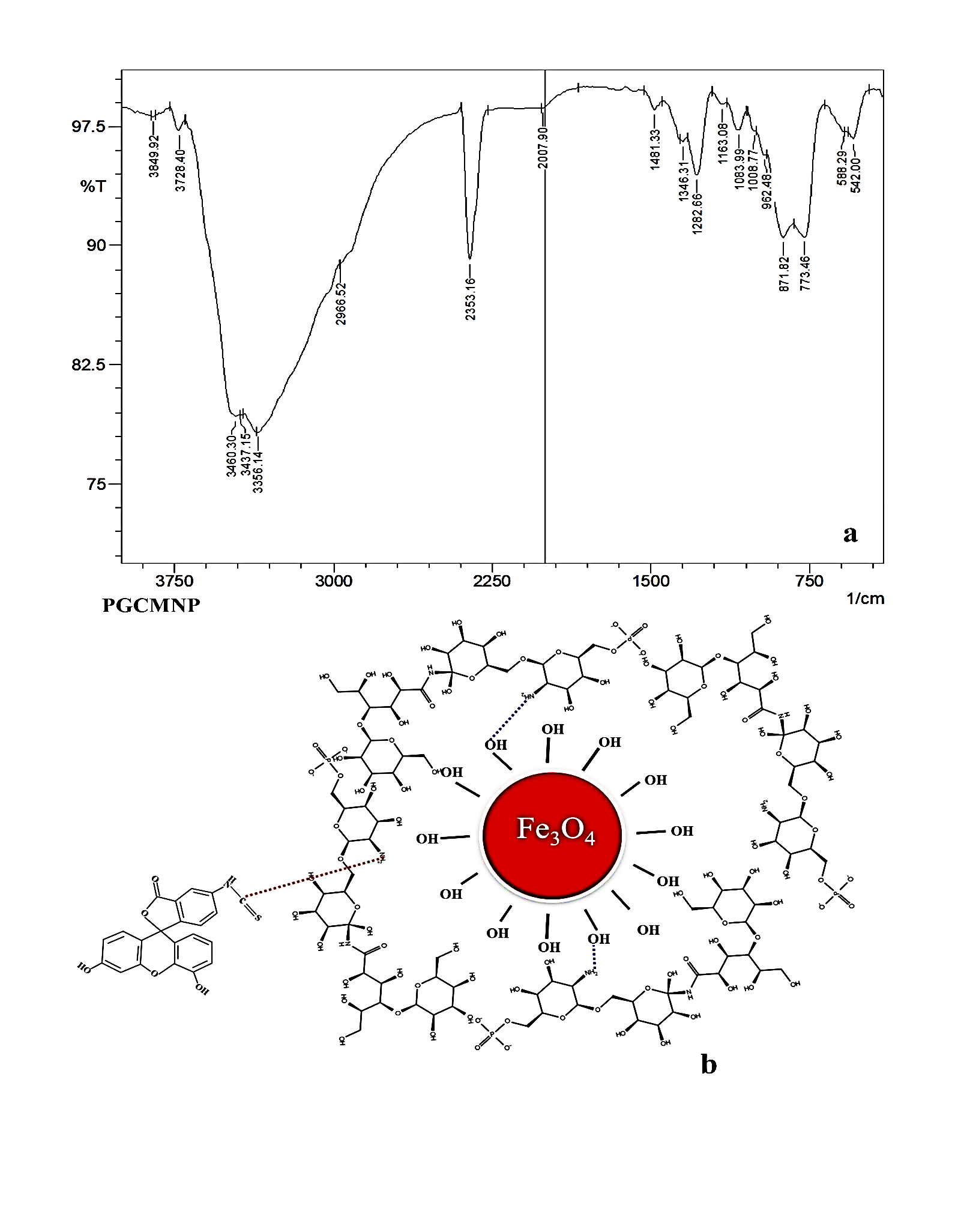
In the FT-IR of FITC tagged PGCMNP, the peak at 2061 cm^-1^ does not appear in the spectrum of PGCMNP+FITC (Fig. 2).

**Figure 2. (a) FT-IR spectra of FITC tagged PGCMNPs; (b) Probable structure of FITC tagged PGCMNPs.**

**Supplementary data 3.**
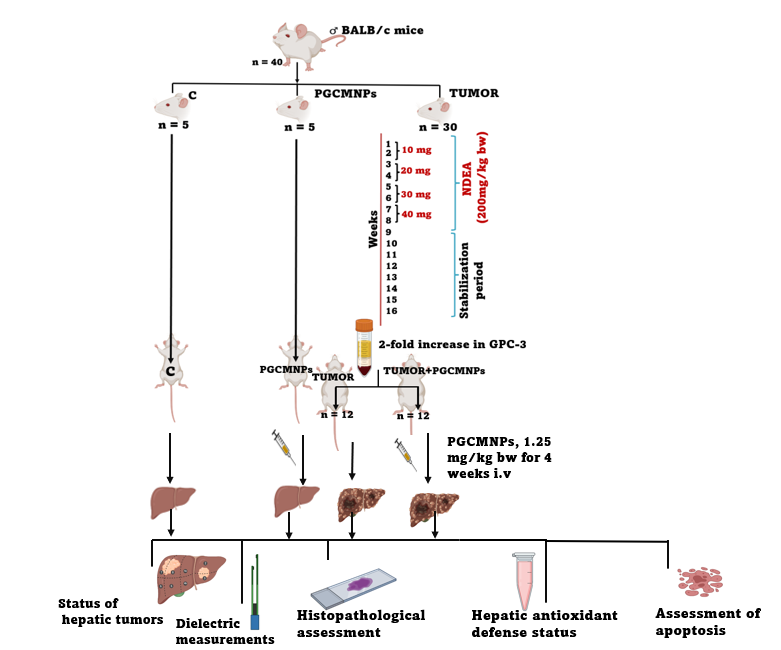
 **Schematic diagram for the development of HCC and PGCMNPs treatment schedule**

**Figure 3. The schematic diagram: In vivo anticancer therapeutic potential of PGCMNPs:** 40 mice were randomly divided into 4 groups: Control (n=5), PGCMNPs (n=5), TUMOR (n=15) and TUMOR+PGCMNPs (n=15). The animals in TUMOR and TUMOR+PGCMNPs groups were given NDEA intraperitoneally for a period of 8 weeks. After 8 weeks, animals with two-fold increase in serum GPC3 levels were chosen. The animals in the PGCMNPs and TUMOR+PGCMNPs groups were treated with PGCMNPs (1.25 mg/kg bw/week for 4 weeks). After 4 weeks of treatment, all the animals were sacrificed, and status of hepatic tumors were noted. Dielectric properties and antioxidant defense status were determined, and tumors were histopathologically assessed for morphological changes and apoptosis.

**Supplementary data 4: PGCMNPs appearance and solubility**

| **Solvent** | **Solubility of PGCMNPs (%)** |
| --- | --- |
| Acetic acid | 97.35 ± 0.180 |
| Sodium hydrogen carbonate | 90.96 ± 1.755 |
| Distilled water | 94.30 ± 0.173 |

PGCMNPs obtained using co-precipitation method of synthesis was dark brown in color. The solubility results of PGCMNPs in acetic acid, sodium hydrogen carbonate and double distilled water were shown in Table 1. PGCMNPs exhibited a good solubility at neutral/physiological pH.

**Table 1. Solubility of PGCMNPs in various solvents.** PGCMNPs exhibited good solubility at physiological pH. Results were expressed as mean ± SD (n=3).

**Supplementary data 5. Storage stability of PGCMNPs**

Storage stability of PGCMNPs was evaluated at different time points (0, 15, 60 days) and different temperatures (-20 °C, 0 °C, 4 °C). At all these time points and storage temperatures the stability of the compound was assessed in terms of change of their appearance, viscosity, sedimentation volume, moisture content , solubility and pH.

At the storage temperature of -20 °C, PGCMNPs displayed no change in the appearance (as dark brown crystalline compound), odour, moisture content, solubility, viscosity and pH upto 15 days. Significant change (p ≤ 0.05) in the sedimentation volume of PGCMNPs was noted on day 15 (0.5) compared to day 0 (1.0). The decreased sedimentation volume of PGCMNPs solution can be attributed to the aggregation of PGCMNPs on standing, which could be resolved with sonication or agitation prior to usage. However, in the case of PGCMNPs stored at the temperatures of 0 °C and 4 °C, significant changes (p ≤ 0.05) in all the parameters were noted on 15^th^ day compared to 0^th^ day (Table 2). Considering this, with the present assessment, PGCMNPs were found to be stable at -20 °C for 15 days.


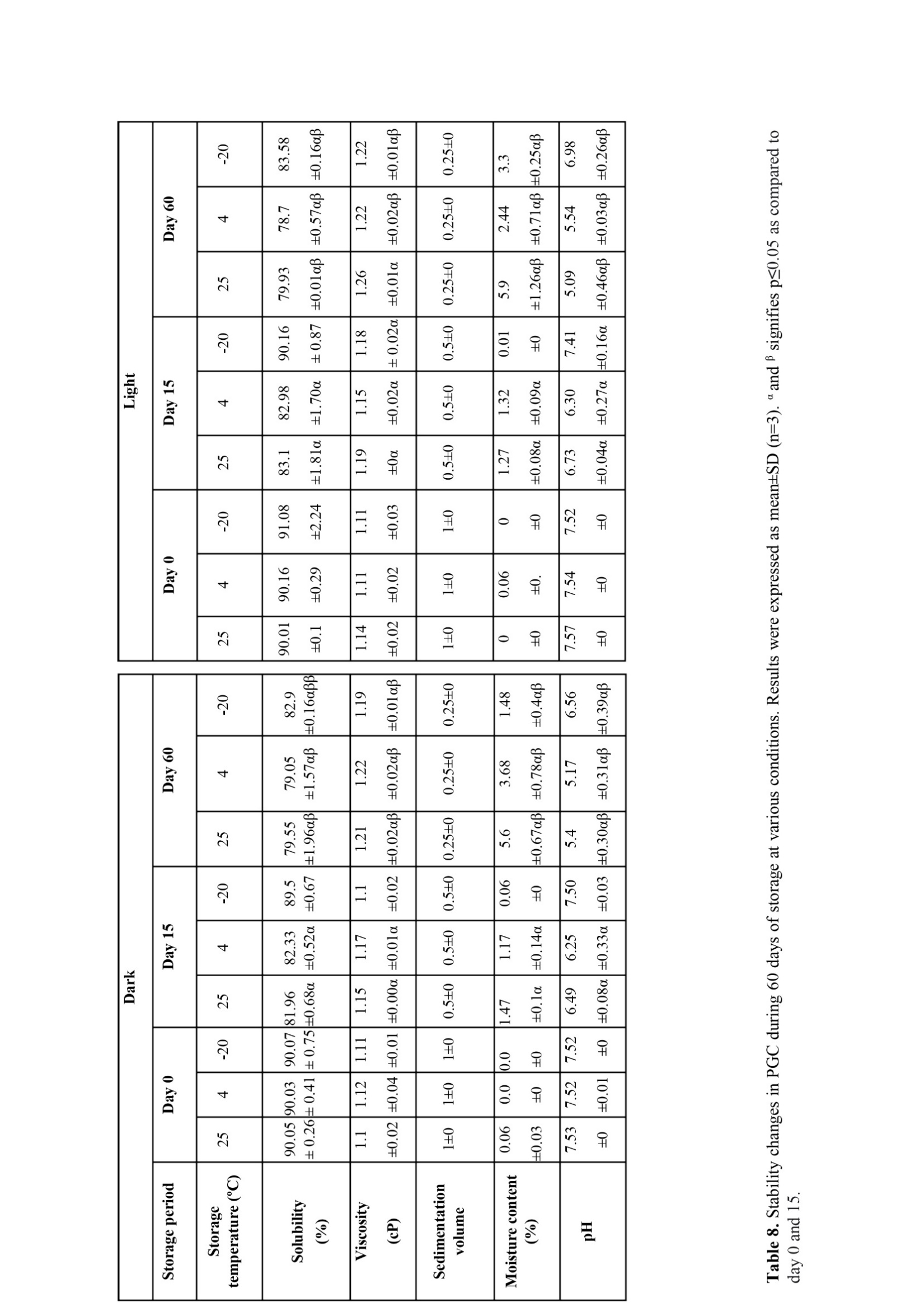


**Table 2.** Stability changes in PGCMNPs during 60 days of storage at various conditions. Results were expressed as mean±SD (n=3). ^α^ and ^β^ signifies p**≤**0.05 as compared to day 0 and 15.

**Supplementary data 6. Nanoparticle tracking analysis of PGCMNPs**


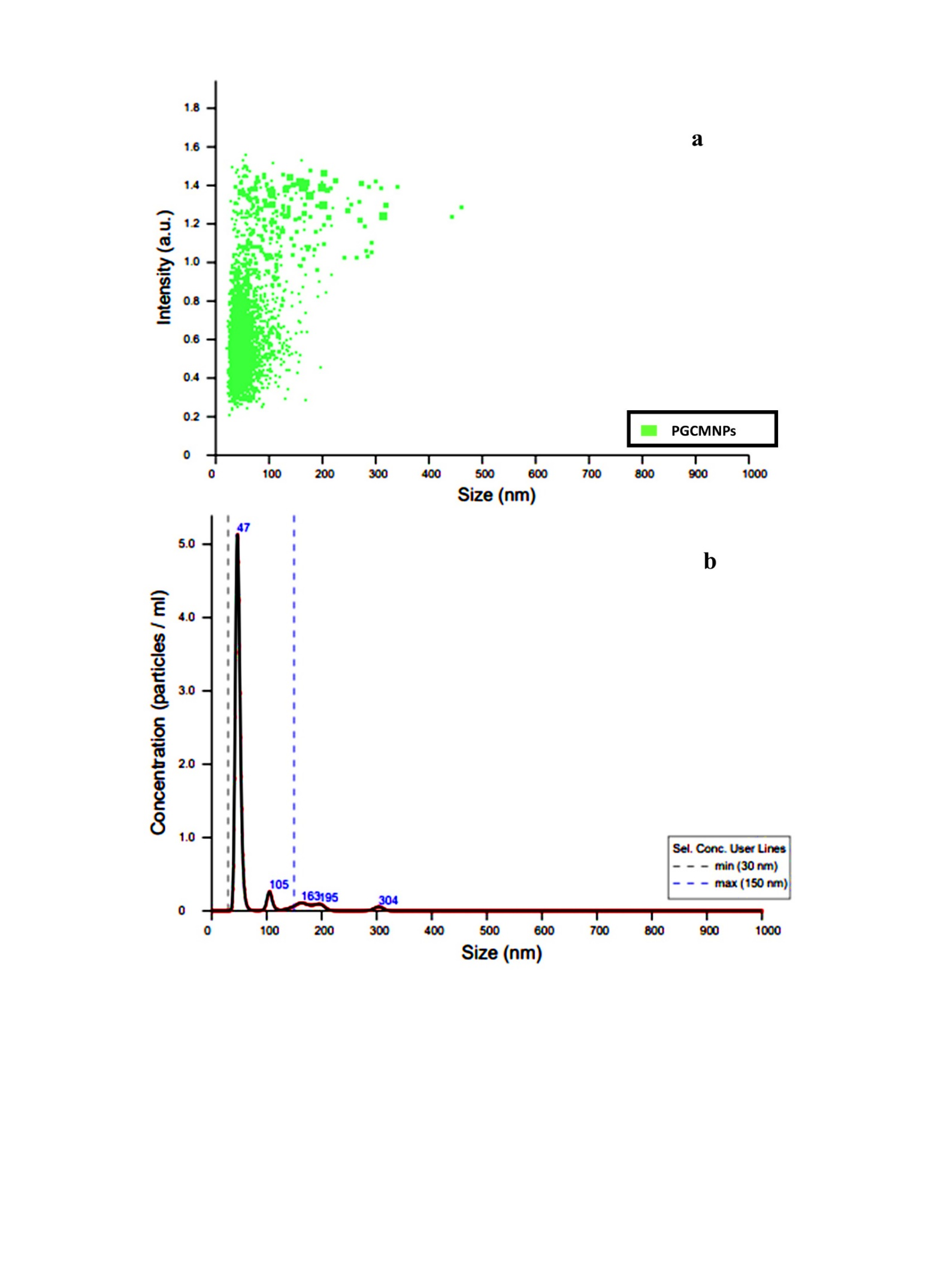
The size distribution and concentration graphs of PGCMNPs also revealed a relatively narrow range of size distribution for the resultant nanoparticles ( Fig. 4a and Fig. 4b ). Narrow range of size distribution was observed with maximum number of particles (90% of the particles) <100 nm.

**Figure 4. Nanoparticle tracking analysis of PGCMNPs.** **(a)** Intensity graph of PGCMNPs **(b)** Concentration of PGCMNPs based on the size. Data was processed using Nanosight software (NTA 3.4 Build 3.4.003, Malvern Panalytical, Malvern, UK).

**Supplementary data 7. Thin layer chromatography of PGCMNPs**


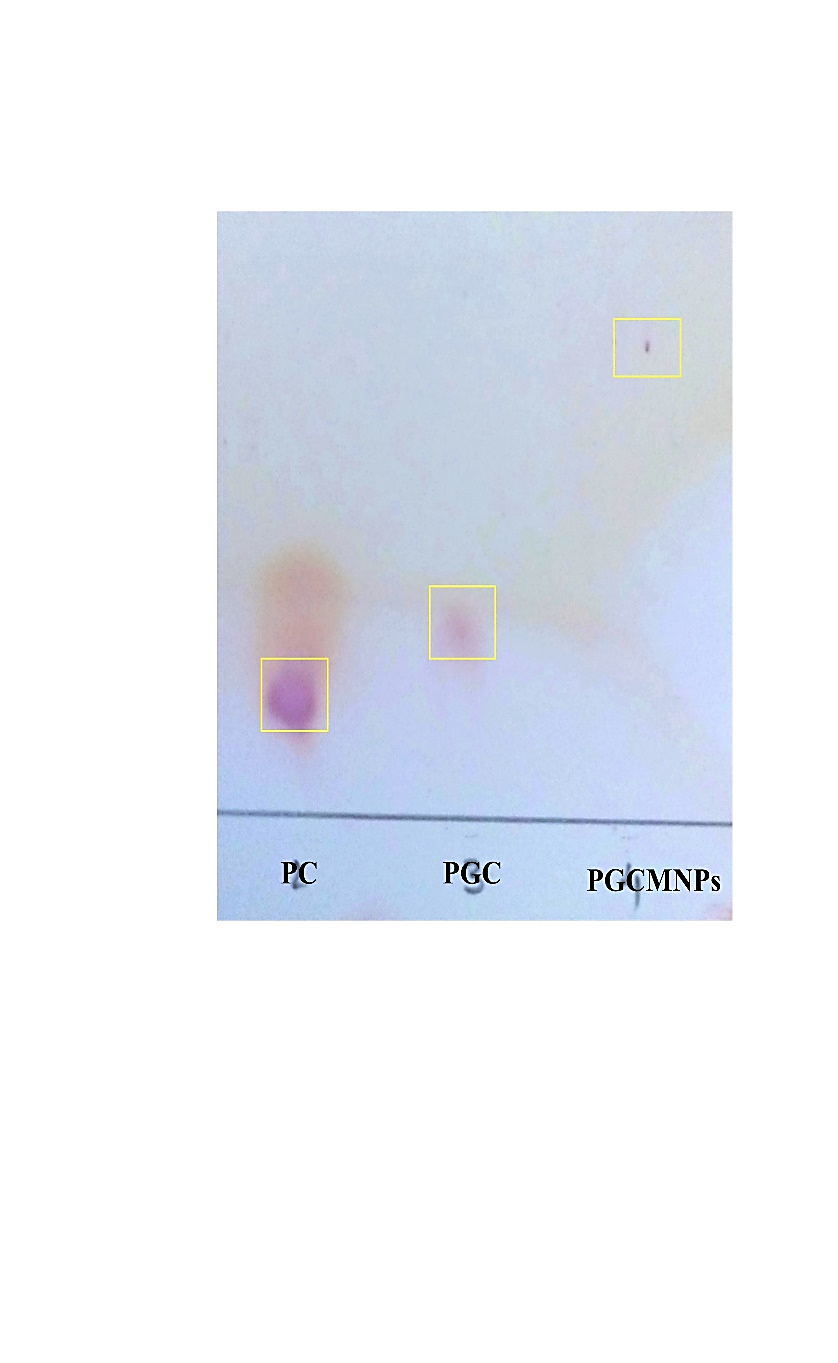
The purity of PGCMNPs was confirmed by thin layer chromatography (Fig. 5). PGC presented big purple spot and PGCMNPs presented small purple spot at different positions on the plate. This indicated that PGC was successfully grafted onto PGCMNPs, and no free groups were present in PGCMNPs.

**Figure 5. Thin layer chromatography analysis of phosphorylated galactosylated chitosan coated magnetic nanoparticles.** **PC:** Phosphorylated chitosan; **PGC:** Phosphorylated galactosylated chitosan; **PGCMNPs:** Phosphorylated galactosylated chitosan coated magnetic nanoparticles. Square represents chromatographic spots of the corresponding compounds.

**Supplementary data 8. Biodistribution of PGCMNPs**


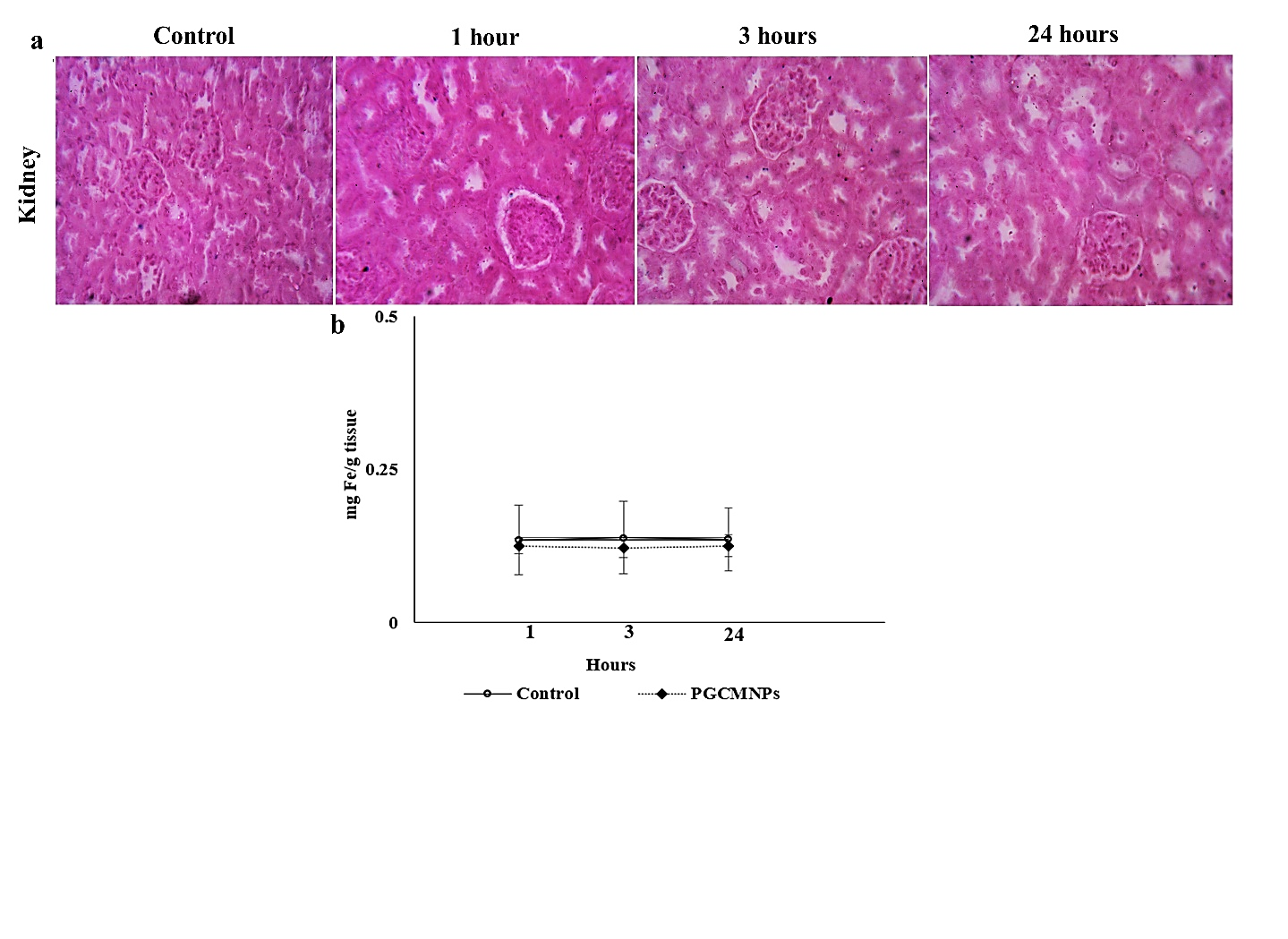
The tissues such as kidney, heart, lung, testis, brain, spleen and intestine did not show any increase in iron levels at each time after the intravenous administration of PGCMNPs (Fig…-Fig…). Therefore, these results indicated that liver was the major target organ for PGCMNPs deposition.

**Figure 6. Biodistribution of PGCMNPs in the kidney. (a)** Representative photomicrographs of kidney stained with Prussian blue to detect iron deposits at 1 hour, 3 hour and 24 hours after the intravenous administration of PGCMNPs (400X) **(b)** Changes in the Fe levels of kidney at 1 hour, 3 hours and 24 hours after the intravenous injection of PGCMNPs.


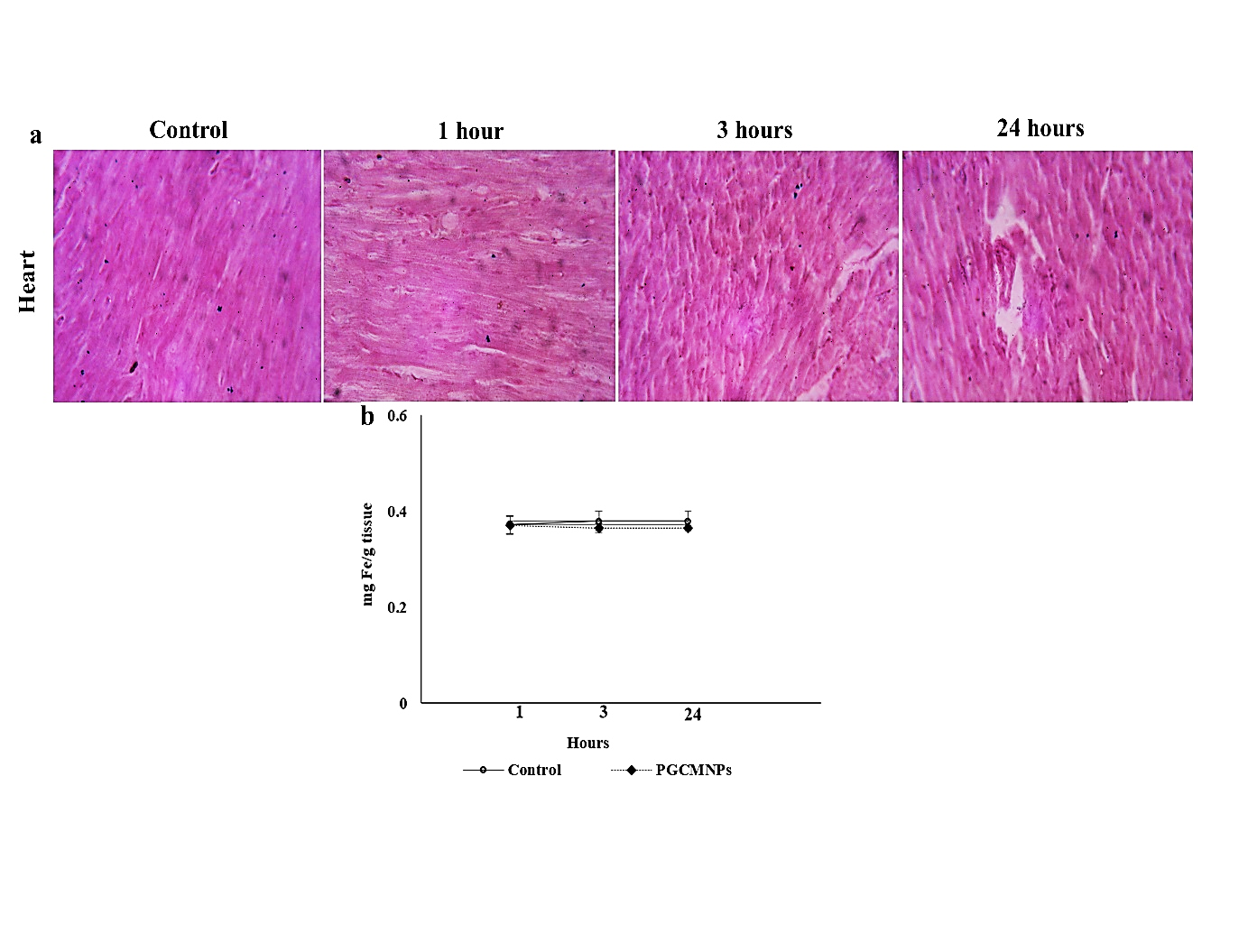


**Figure 7. Biodistribution of PGCMNPs in the heart. (a)** Representative photomicrographs of cardiac tissue stained with Prussian blue to detect iron deposits at 1 hour, 3 hour and 24 hours after the intravenous administration of PGCMNPs (400X) **(b)** Changes in the Fe levels of heart at 1 hour, 3 hours and 24 hours after the intravenous injection of PGCMNPs.


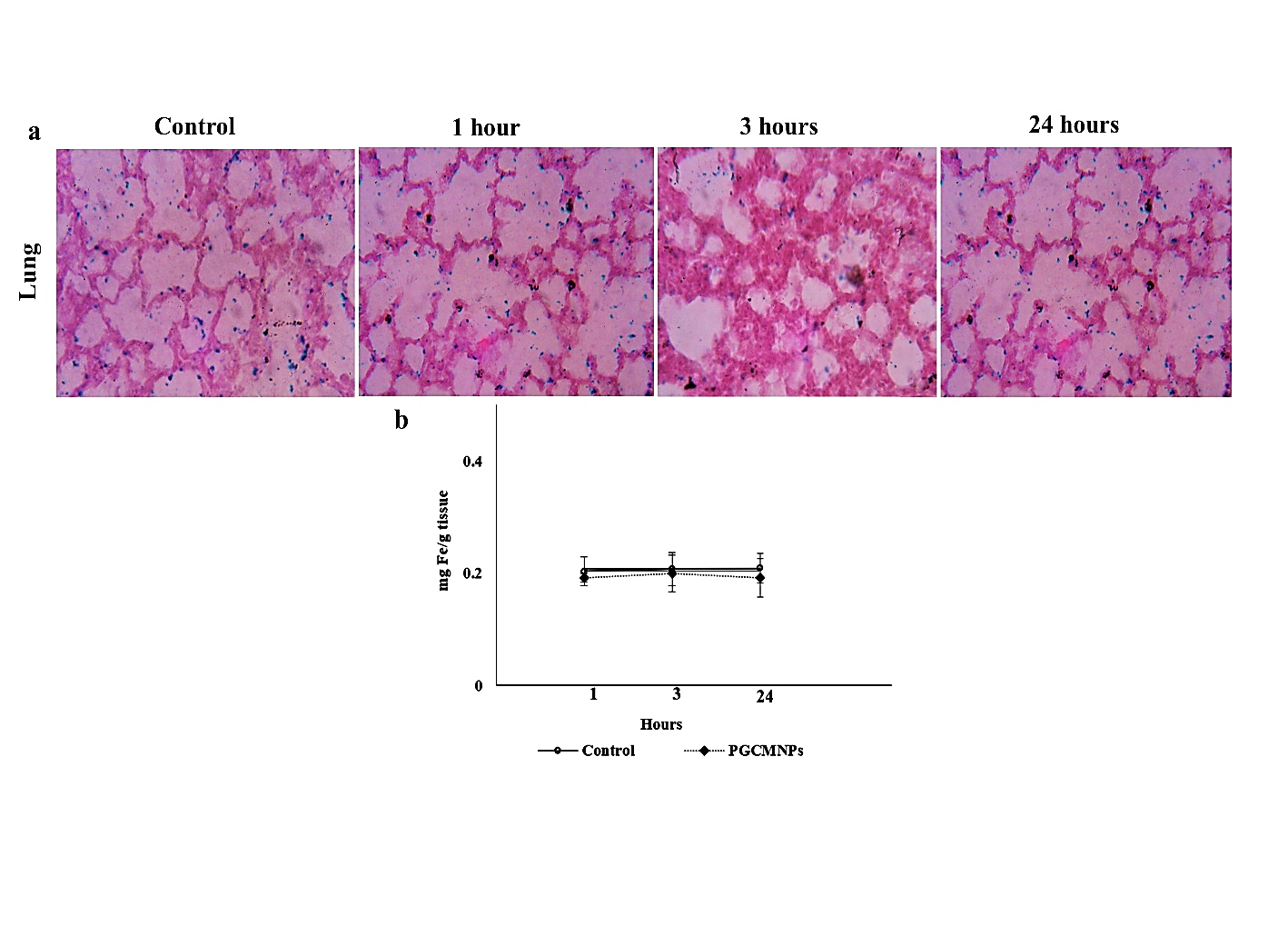
**Figure 8. Biodistribution of PGCMNPs in the lung. (a)** Representative photomicrographs of lung tissue stained with Prussian blue to detect iron deposits at 1 hour, 3 hour and 24 hours after the intravenous administration of PGCMNPs (400X) **(b)** Changes in the Fe levels of lung at 1 hour, 3 hours and 24 hours after the intravenous injection of PGCMNPs.


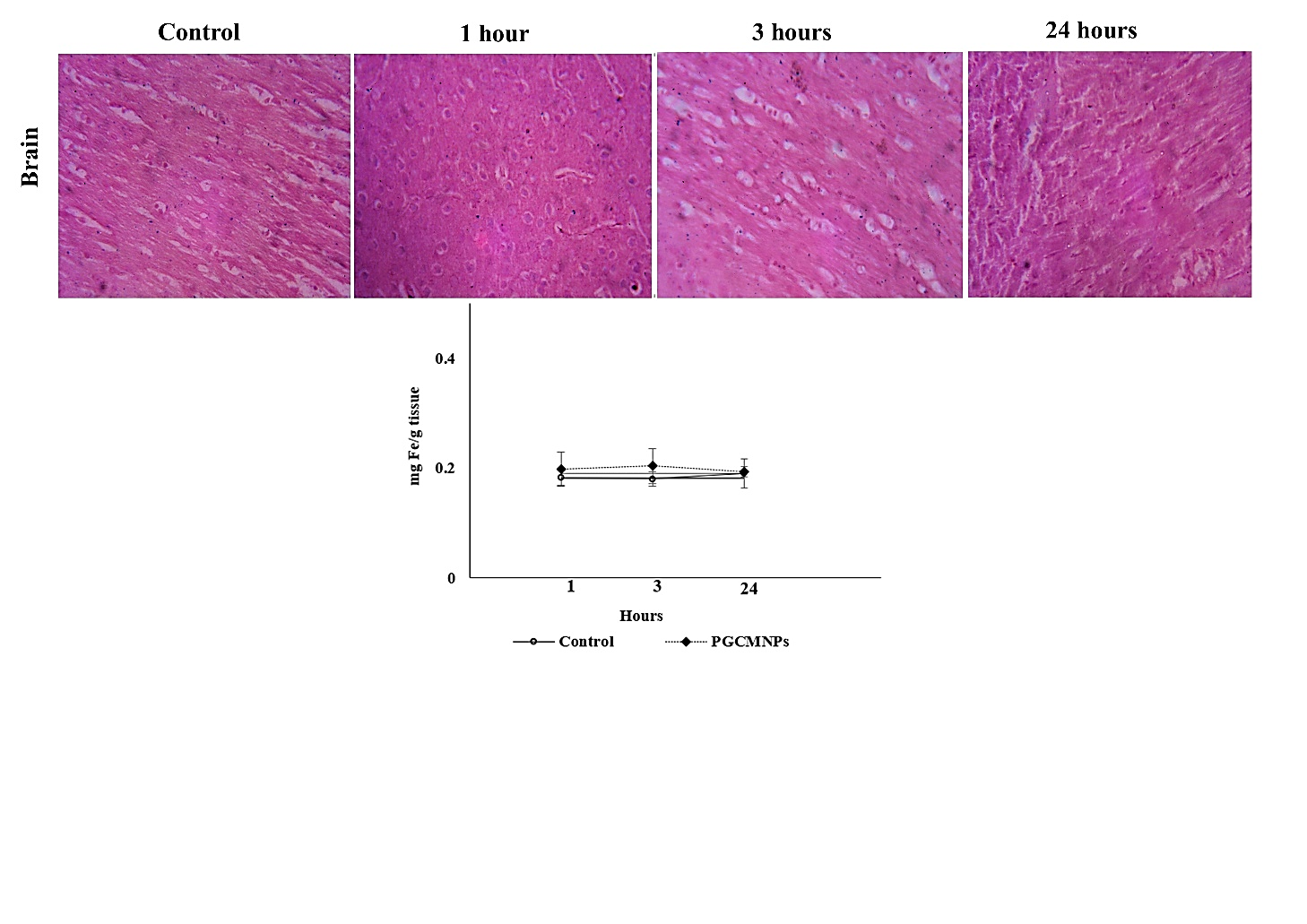
**Figure 9. Biodistribution of PGCMNPs in the brain. (a)** Representative photomicrographs of brain stained with Prussian blue to detect iron deposits at 1 hour, 3 hour and 24 hours after the intravenous administration of PGCMNPs (400X) **(b)** Changes in the Fe levels of brain at 1 hour, 3 hours and 24 hours after the intravenous injection of PGCMNPs.


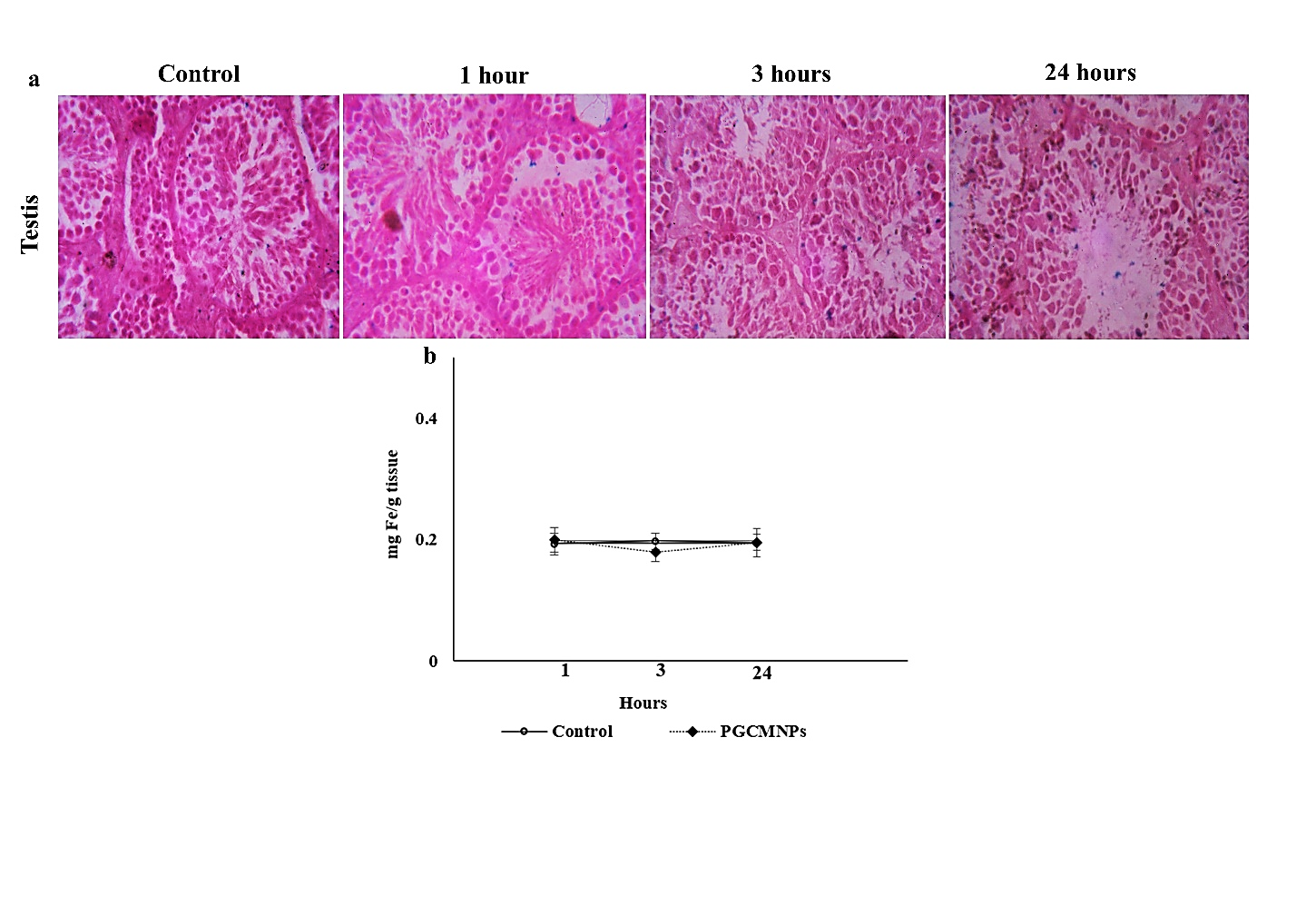
**Figure 10. Biodistribution of PGCMNPs in the testis. (a)** Representative photomicrographs of testis stained with Prussian blue to detect iron deposits at 1 hour, 3 hour and 24 hours after the intravenous administration of PGCMNPs (400X) **(b)** Changes in the Fe levels of testis at 1 hour, 3 hours and 24 hours after the intravenous injection of PGCMNPs.


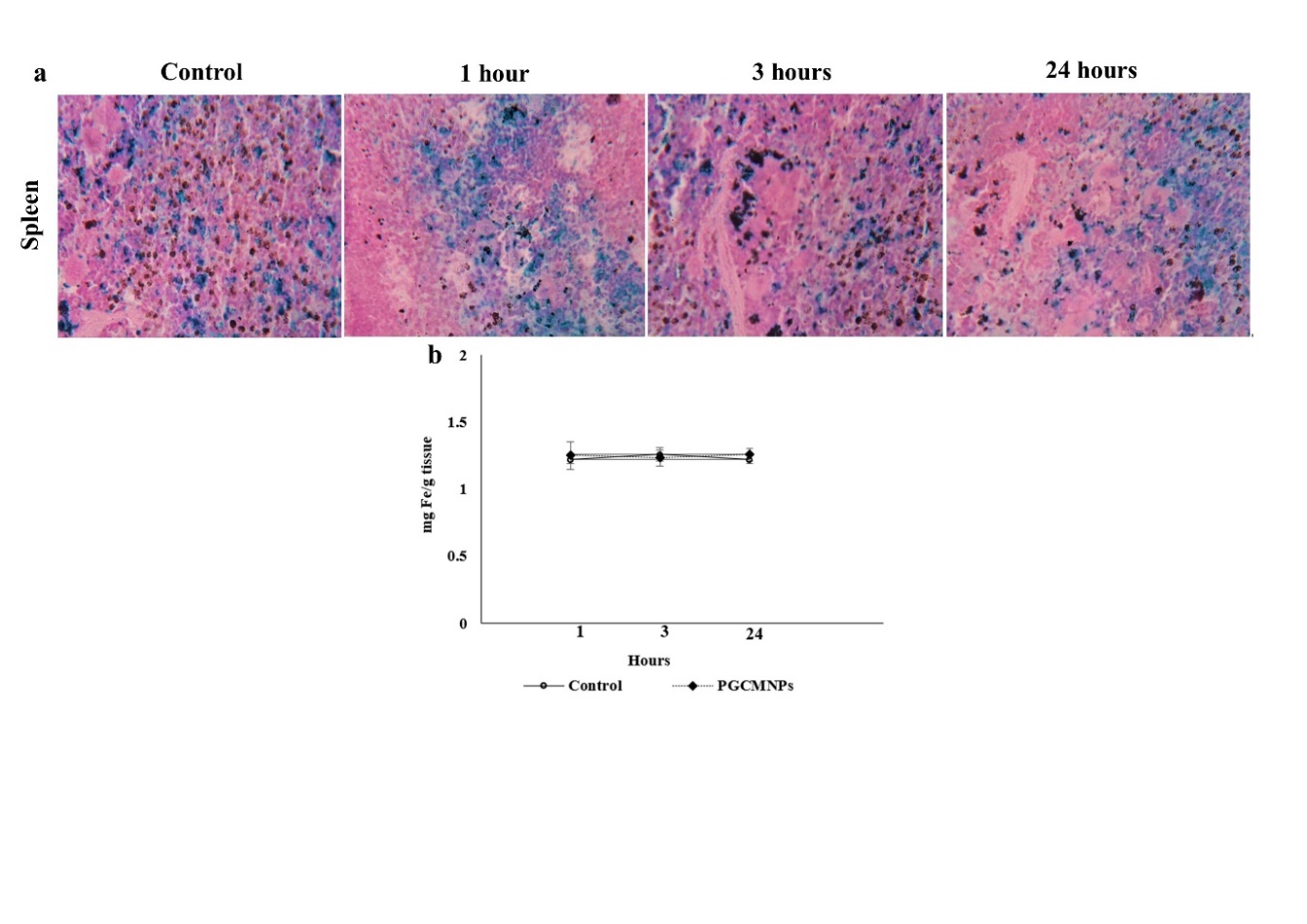


**Figure 11. Biodistribution of PGCMNPs in the spleen. (a)** Representative photomicrographs of spleen stained with Prussian blue to detect iron deposits at 1 hour, 3 hour and 24 hours after the intravenous administration of PGCMNPs (400X) **(b)** Changes in the Fe levels of spleen at 1 hour, 3 hours and 24 hours after the intravenous injection of PGCMNPs.


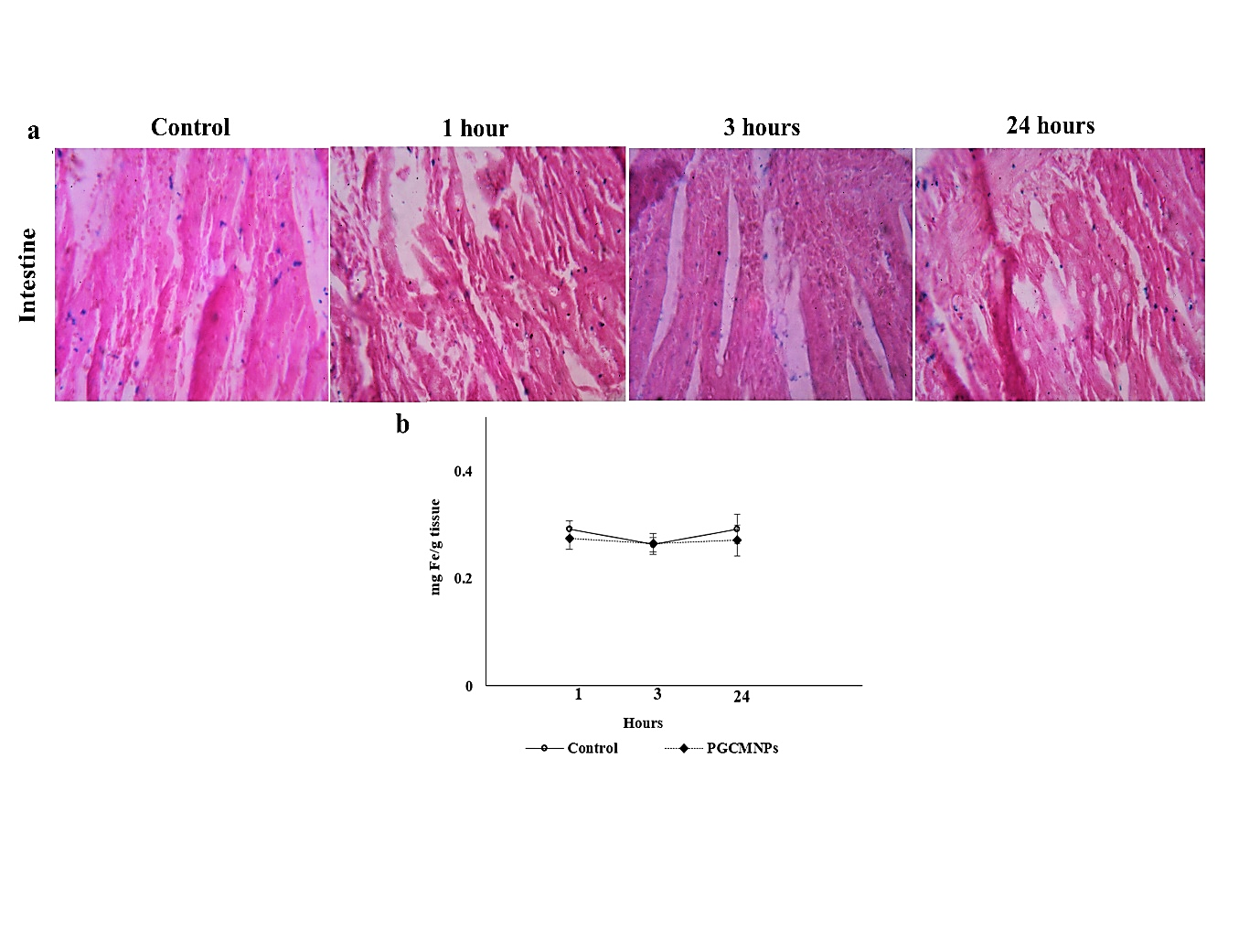


**Figure 12. Biodistribution of PGCMNPs in the intestine. (a)** Representative photomicrographs of intestine stained with Prussian blue to detect iron deposits at 1 hour, 3 hour and 24 hours after the intravenous administration of PGCMNPs (400X) **(b)** Changes in the Fe levels of intestine at 1 hour, 3 hours and 24 hours after the intravenous injection of PGCMNPs.
